# Crowdsourcing bridge dynamic monitoring with smartphone vehicle trips

**DOI:** 10.1038/s44172-022-00025-4

**Published:** 2022-11-03

**Authors:** Thomas J. Matarazzo, Dániel Kondor, Sebastiano Milardo, Soheil S. Eshkevari, Paolo Santi, Shamim N. Pakzad, Markus J. Buehler, Carlo Ratti

**Affiliations:** 1 Senseable City Laboratory, MIT, Cambridge, MA 02139 USA; 2 Cornell Tech, Cornell University, New York, NY 10044 USA; 3 Dept. of Civil and Mechanical Engineering, United States Military Academy, West Point, NY 10996 USA; 4 Center for Innovation and Engineering, United States Military Academy, West Point, NY 10996 USA; 5 Singapore-MIT Alliance for Research and Technology, Singapore, Singapore; 6 Dept. of Civil and Environmental Engineering, Lehigh University, Bethlehem, PA 18015 USA; 7 Istituto di Informatica e Telematica del CNR, Pisa, Italy; 8 Dept. of Civil and Environmental Engineering, Massachusetts Institute of Technology, Cambridge, MA 02139 USA

**Keywords:** Civil engineering, Scientific data, Information technology, Mechanical properties

## Abstract

Monitoring and managing the structural health of bridges requires expensive specialized sensor networks. In the past decade, researchers predicted that cheap ubiquitous mobile sensors would revolutionize infrastructure maintenance; yet extracting useful information in the field with sufficient precision remains challenging. Herein we report the accurate determination of critical physical properties, modal frequencies, of two real bridges from everyday vehicle trip data. We collected smartphone data from controlled field experiments and uncontrolled Uber rides on a long-span suspension bridge in the USA (The Golden Gate Bridge) and developed an analytical method to accurately recover modal properties. We also successfully applied the method to partially-controlled crowdsourced data collected on a short-span highway bridge in Italy. Further analysis projected that the inclusion of crowdsourced data in a maintenance plan for a new bridge could add over fourteen years of service (30% increase) without additional costs. Our results suggest that massive and inexpensive datasets collected by smartphones could play a role in monitoring the health of existing transportation infrastructure.

## Abstract (web summary)

Thomas Matarazzo and colleagues determine modal frequencies of a suspension bridge and highway bridge from analysis of smartphone datasets during vehicle trips across the bridge. The field results suggest that data from both controlled and uncontrolled crowdsourced smartphone datasets have value in monitoring transportation infrastructure.

## Introduction

Mobile sensors could reform the way we measure infrastructure health. Smartphones contain dozens of sensors that are carried by almost 50% of the population globally^1^. Analyses of crowdsourcing networks have uncovered truths about the social, economical, civil, and technological systems we rely on in an urban environment, e.g., quantifying urban human mobility^2–4^, understanding the perception of built environment^5^, modeling and predicting infectious disease spread^6^, etc.^7–9^. Recently, there has been an increased focus on self-sustaining sensing platforms such as data generated by vehicle fleets, either with smartphones or dedicated sensors^10–12^.

The effectiveness of a crowdsourcing application is a question of precision and scale. While crowdsourced data has a proven value and cost-effectiveness in a variety of large-scale applications, there remain fundamental challenges for those that call for precise measurements in time and space. Civil infrastructure monitoring techniques require highly curated data, often sampled at a high rate by synchronous data-acquisition systems and low noise sensors. Nearly two decades ago, the potential of mobile-sensor networks was established analytically and using synthetic models^13–15^. Following this foundational work, researchers have been eager to produce successful real-world applications that use crowdsourced smartphone data to extract the dynamical properties of existing bridges – a problem with stringent spatiotemporal measurement constraints.

There is a global need to endorse infrastructure monitoring to optimize the service of the most critical assets of highway networks and the urban environment. A failure to maintain a city’s bridges, buildings, and other infrastructure can have disastrous consequences, from enormous repair costs to the loss of human life^16^. The vast number of U.S. bridges with structural problems accentuates shortcomings in bridge maintenance protocols^17,18^. Modern bridge condition assessments are based on field inspection notes from visual inspections rather than large digital datasets; a paradigm that severely limits the frequency of structural health assessments, the depth of the information collected, and the ability to execute preventive maintenance.

Crowdsourcing bridge vibration data would modernize structural health monitoring (SHM) and bridge asset management at a global scale. Longitudinal data collection and analyses are essential for tracking changes in structural state, informing preemptive repairs, and service life analyses^19–25^. In typical SHM applications, a synchronized sensor network is mounted on a bridge (scale on the order of hectometers to kilometers) to measure acceleration (scale on the order of millimeters per second squared (milli-*G*)). While sensor data provides advantages over field inspections, due to high costs, such static sensor networks are rarely incorporated in a bridge management system. Despite the implications of Moore’s Law, installation and maintenance costs are often still too high for the vast majority of bridge owners.

Mobile-sensor networks resolve this financial bottleneck by bypassing high-end sensing systems. Smartphones or other cheap sensors, either mounted on^11^ or riding in vehicles^12^, can contribute useful data. The primary appeal of a mobile-sensor network is that it does not require dedicated devices: it can repurpose existing ones resulting in cheaper and more convenient data collection compared to traditional methods. For example, smartphones can scan a city’s infrastructure with a wide spatiotemporal coverage at little or no cost through the existing travel patterns of the host vehicles, e.g., taxis^12^. A recent study showed that just two mobile sensors produces SHM information comparable to 240 static sensors^26^, with other studies reporting similarly large efficiencies^27–32^. Simultaneously, applications of deep learning in material science have exemplified how sparse structural response measurements can be used to accurately predict global behavior, e.g., load distributions^33^, and optimize multiscale design.

Recent applications of smartphones in civil engineering^34–38^ showed that while the embedded accelerometers exhibit many undesirable characteristics, they can sufficiently capture structural vibrations. Simultaneously, theoretical and experimental research on vehicle-bridge interaction relationships have established governing equations and influential parameters^36,39–45^. Nevertheless, there remain open questions on estimation precision in a real setting. For instance, the literature lacks a successful example in which bridge modal properties are extracted exclusively from crowdsourced mobile smartphone data. Most importantly, prior work has not considered uncontrolled or partially controlled smartphone datasets (from ridesourcing or crowdsourcing), i.e., cases where analysts have little or no influence over key data collection parameters (see “Methods” for detailed classifications of data controllability).

As such, the core question remains unanswered: *can crowdsourcing produce precise structural health information under real-world conditions?* This paper provides strong evidence that supports crowdsourcing. It shows that data collected by smartphones in moving vehicles under real-world conditions can be used to identify structural modal properties of a bridge, information which is vital to condition assessments and damage detection frameworks^13,20,23–25,46,47^. The following results based on uncontrolled and partially controlled crowdsensed data collected on two structurally diverse bridges, verify that pre-existing mobile sensing mechanisms and datasets, originally established for other purposes, can produce important structural information and therefore could be utilized to extensively monitor the health of networks of bridges, worldwide.

## Results and discussion

### Determining most probable modal frequencies

The Golden Gate Bridge is a long-span suspension bridge in California, USA, with a 1280-meter-long main span. Two distinct datasets were collected: a field study where researchers drove over the bridge 102 times, recording data with two smartphones (iPhone 5 and iPhone 6) and data collected by Uber drivers in 72 bridge trips during normal operations. In the following, these are referred to as controlled and ridesourcing datasets. Impressively, while these datasets are relatively small, they produce accurate results, demonstrating that mobile-sensor-based SHM can be applied easily, cheaply, and immediately in the real world.

**Fig. 1:**
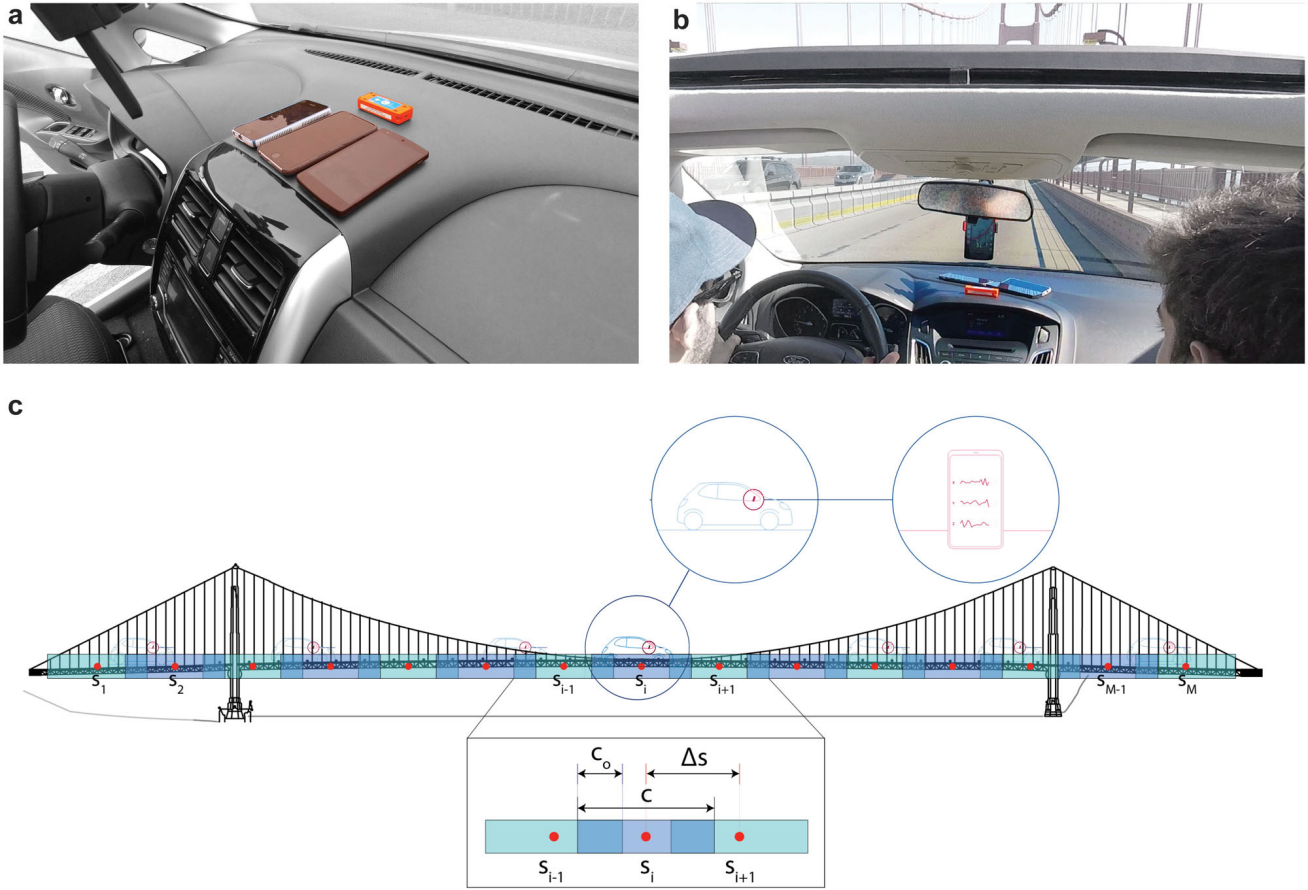
Illustration of controlled data collection and the spatial segmentation approach. **a** Sensor layout on the dashboard of the first vehicle (Nissan Sentra) which was used to collect first fifty trips. **b** Sensor layout on dashboard of the second vehicle (Ford Focus) which was used to collect fifty-two trips. For all vehicle trips over the Golden Gate Bridge, The smartphones were facing upward, such that one axis was well-aligned with gravity. Such an orientation is not strictly necessary; although, knowledge on the configuration of the sensors is helpful for data preprocessing. **c** Generic schematic of spatial segmentation of a bridge which is defined through two independent parameters: Δ*s* and *c*, which remain uniform over the length of the bridge. The red circles represent the centers of each segment, while the light colored boxes show the segment widths. A close-up of three adjacent segments *s*_*i*−1_, *s*_*i*_, and *s*_*i*+1_, is shown to detail the segmentation parameters: *c* is the length of each segment, *c*_*o*_ is the length of the overlap between segments, and Δ*s* is the distance between the centers (red circles) of adjacent segments.

Figure 1 describes the controlled data collection process and the spatial analysis method. The experiments focus on the identification of the first ten vertical and torsional frequencies of the Golden Gate Bridge, which are below 0.5 Hz (see “Methods” for a complete catalog).

**Fig. 2:**
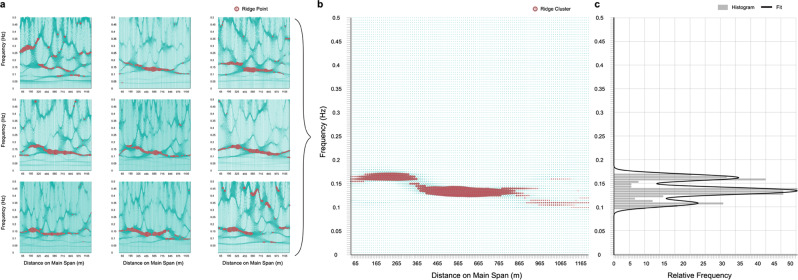
Illustration of the main methodology used to extract most probable modal frequencies (MPMFs). **a** The synchrosqueezed wavelet transform is calculated for each of the bride crossings individually. The time variable is then remapped into linear location (*r*) on the bridge, resulting in the space-frequency representation of the signal. Ridges are then identified as peaks at each location. (Steps 1-6 in the methodology, see “Methods”). **b** Peaks from each individual location are first aggregated in the spatial groups shown in Fig. 1, then among all datasets, resulting in one space-frequency diagram of identified ridge clusters. Each location in space represents one spatial segment from Fig. 1 (Step 7). **c** The most prominent vibration frequencies from each spatial group are selected and a histogram of these is created. The modes of this histogram are identified using a kernel density (KDE) fit; these picks are considered the MPMFs (Steps 8–9 in methodology, see “Methods”).

The methodology developed to determine the most probable modal frequencies (MPMFs), the main result of the analysis, is illustrated in Fig. 2. The plots in Fig. 2a are produced via the synchrosqueezed wavelet transform^48^ (with Morlet basis) and a mapping of time to a local coordinate system (in space) based on simultaneous Global Positioning System (GPS) measurements. Figure 2b shows the data aggregation step: a plot of the frequencies that were most consistently present over all the trips versus bridge length (space). In the final step, depicted in Fig. 2c, a kernel density estimate (KDE) is fit to the histogram (displayed as solid line) of the frequency candidates. Further details are presented in the “Methods” section and Supplementary Notes 1 and 2.

**Fig. 3:**
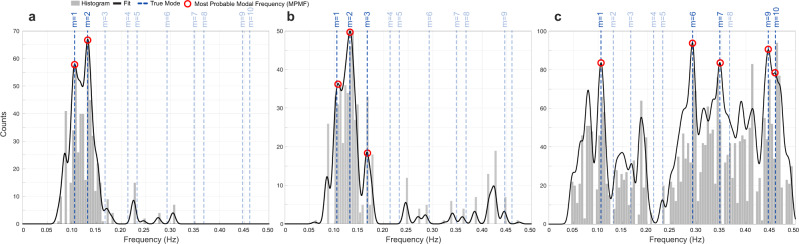
Final estimates of probability density functions (PDFs) from smartphone vehicle trip (SVT) data. A histogram (gray bars) and kernel density estimates (black line, Gaussian kernel with bandwidth of 0.005 Hz) is shown for each dataset: (**a**) results from iPhone 5 in controlled data. (**b**) Results from iPhone 6 in controlled data, (**c**) results from ridesourcing data. Blue dashed lines refer to true modal frequencies of the bridge (see Table 1). The local maxima (modes) of each multimodal PDF corresponds to a possible modal frequency. The iPhone 5 most probable modal frequencies (MPMFs) correspond to the two largest peaks, which estimate the first and second modal frequencies (*m* = 1, 2). The iPhone 6 MPMFs correspond to the three largest peaks, which estimate the first, second, and third modal frequencies (*m* = 1, 2, 3). The known modal frequencies of the Golden Gate Bridge are indicated for reference. Details of the MPMFs displayed in (**a**) and (**b**) are provided in Table 1. In the PDFs based on ridesourcing data there are over a dozen MPMF candidates (local maxima), a significance threshold of 0.90 was set for the CDF values, which resulted in five MPMFs. Details of the MPMFs displayed in (c) are provided in Table 2.

The MPMFs are defined as the peaks of the KDE probability density function. The MPMF results for the controlled data and ridesourcing data and their corresponding probability density functions are displayed in Fig. 3; these plots highlight the likely vibrational frequencies. The MPMF values are displayed in Tables 1 and 2 for the controlled and ridesourcing data, respectively. For KDE, a Gaussian kernel was selected with a bandwidth equal to 1% of the frequency range considered (0.005 Hz since the range is 0–0.5Hz). Initially, MPMFs were chosen by visual inspection of the PDF. The corresponding cumulative distribution function (CDF) indicated that the chosen MPMFs for the iPhone 5 were in the upper 2% and those MPMFs for the iPhone 6 were in the upper 10% (see Table 1 for precise values). That is for all MPMFs in this work, the selection was automated by setting an upper threshold for the CDF of the frequency candidates, e.g., 10%.

**Table 1:** Most probable modal frequencies (MPMFs) for the controlled data, extracted from the peaks of Fig. 3a, b.

	iPhone 5		iPhone 6		
*m*	MPMF	CDF	MPMF	CDF	True
1	0.106 (0.000)	0.98	0.108 (1.89)	0.95	0.106
2	0.132 (0.000)	1.00	0.132 (0.000)	1.00	0.132
3	–	–	0.166 (2.35)	0.90	0.170

The vibration mode numbers are indicated by m, where frequencies are sorted in ascending order. MPMFs (estimates) and true frequency values are in Hertz with corresponding errors (%) in parentheses. Cumulative distribution function (CDF) values indicate the significance of each peak in the overall probability density function (PDF).

**Table 2:** Most probable modal frequencies (MPMFs) for the ridesourcing data, were extracted as the peaks of Fig. 3c in the top ten percentile (five in total).

*m*	MPMF	CDF	True
1	0.106 (0.000)	0.97	0.106
6	0.291 (3.32)	1.00	0.301
7	0.347 (2.35)	0.97	0.339
9	0.445 (0.000)	0.99	0.445
10	0.458 (0.650)	0.93	0.461

The vibration mode numbers are indicated by m, where frequencies are sorted in ascending order. MPMFs (estimates) and true frequency values are in Hertz with corresponding errors (%) in parentheses. Cumulative distribution function (CDF) values indicate the significance of each peak in the overall probability density function (PDF).

The MPMF results are compared with the most comprehensive report on the modal properties of the Golden Gate Bridge. The true values in Tables 1 and 2 are based on data collected over a three-month period with a wireless network of 240 accelerometers and found over sixty vibrational modes (vertical, transverse, and torsional)^49,50^. In particular, there are seven vertical modes and three torsional modes below 0.5 Hz (see Table 3 in “Methods”). The fundamental vibrational modes (lowest frequencies) are often the highest contributors to the overall dynamic response of a structure^51^; i.e., they are most important.

**Table 3:** Ten modal frequencies of the Golden Gate Bridge from ref. 50: seven vertical and three torsional, all below 0.5 Hz.

*m*	Type	Shape	Frequency (Hz)
1	V	A	0.106
2	V	S	0.132
3	V	S	0.170
4	V	A	0.216
5	T	A	0.230
6	V	S	0.301
7	T	S	0.339
8	V	A	0.371
9	T	A	0.445
10	V	S	0.461

The vibration mode numbers are indicated by m, where frequencies are sorted in ascending order; mode types V and T refer to vertical and torsional modes; A and S refer to anti-symmetric and symmetric mode shapes with respect to the middle of the main bridge span.

Overall, the first two modal frequencies of the bridge are estimated accurately by both the iPhone 5 and the iPhone 6 in the controlled experiments (see Fig. 3 and Table 1). The iPhone 5 data estimates the first frequency as 0.106 Hz and the second frequency as 0.132 Hz. Similarly, the iPhone 6 data estimates the first frequency as 0.108 Hz and the second frequency as 0.132 Hz. In addition, the iPhone 6 data estimates the third modal frequency as 0.166 Hz. Estimates made by the iPhone 5 for both frequencies and the iPhone 6 for the second frequency are accurate up to three significant digits, the precision used in our study (<0.5% error). Estimates of the first and third frequency by the iPhone 6 have errors of 1.9% and 2.3% respectively. These errors are low and within an acceptable range in the context of operational modal identification. In practice, independent methods are employed to corroborate estimates of modal properties. For a given dataset, modal frequency estimates will vary based on the method yet will often be within 1−2% of each other^52^; in some cases discrepancies can be as high as 5−7% for certain modes, methods, and datasets^40^.

The ridesourcing data consists of 72 datasets representing 37 different vehicles and 19 smartphone models, which were generally collected at higher driving speeds (see Tables 4 and 5 in “Methods”). In the PDF from the ridesourcing data, there are over a dozen MPMF candidate peaks that are distributed more evenly (see Fig. 3c). The MPMFs were chosen as the peaks in the upper 10% of the CDF, which resulted in five candidates. Table 2 compares these MPMFs with the true modal frequencies. Impressively, each of the five MPMFs corresponds to a true modal frequency and includes the fundamental mode (*m* = 1). Additionally, the MPMFs here include four new modes that were not detected in the controlled trip data (*m* = 6, 7, 9, 10). This result reflects how the content of the frequency information is subject to the context of the vehicle trips, such as, vehicle attributes, smartphone sensors, etc. In general, in order to be measured, the frequency content must be present in the bridge vibrations, meaning the modes of interest must be externally excited by the dynamic loads, e.g., traffic, wind, etc. (see Supplementary Note 1 for further details).

**Table 4:** List of thirty-seven vehicle types present in the ridesourcing dataset.

Vehicle type	Number of trips
BMW 3-series	1
BMW X1	1
Chevrolet Cruze	3
Chevrolet Malibu	2
Chevrolet Trax	2
Chrysler Pacifica	1
Ford Focus	1
Ford Fusion	3
Ford Taurus	1
Honda Accord	3
Honda Civic	2
Honda CR-V	1
Honda Insight	1
Honda Odyssey	1
Hyundai Elantra	2
Hyundai Ioniq	1
Hyundai Sonata	3
Kia Forte	2
Kia Optima	1
Lexus RX	1
Mazda CX-5	3
Mazda CX-9	1
Mazda MAZDA6	1
Mercedes-Benz C-Class	1
Mercedes-Benz E-Class	1
Mitsubishi Outlander	1
Nissan Altima	4
Nissan Frontier	1
Subaru Impreza	1
Subaru Outback	1
Toyota C-HR	1
Toyota Camry	5
Toyota Corolla	2
Toyota Prius	9
Toyota RAV4	1
Toyota Sienna	4
Volkswagen Jetta	2

**Table 5:** List of nineteen smartphone types present in the ridesourcing dataset.

Phone model	Number of trips
iPhone 5s	2
iPhone 6	2
iPhone 6s Plus	2
iPhone 7	3
iPhone 7 Plus	7
iPhone X	2
iPhone 8 Plus	1
Pixel 2	1
Pixel 2 XL	1
Galaxy J7	2
Galaxy Note 4	10
Galaxy Note 8	3
Galaxy Note Edge	5
Galaxy S4	1
Galaxy S5	17
Galaxy S6	4
Galaxy S7	3
Galaxy S8	5
Galaxy S9	1

The fundamental vertical frequency (*m* = 1) and the third torsional frequency (*m* = 9) were estimated perfectly to the nearest thousandth (0.000% error). The fifth vertical frequency (*m* = 6), the second torsional frequency (*m* = 7), and the seventh vertical frequency (*m* = 10), were estimated with errors of 3.3%, 2.3%, and 0.65%, respectively. It is important to note that the proposed method can detect both vertical and torsional modes because the dataset provided vertical (gravity direction) acceleration measurements. In this study, distinctions between vertical and torsional MPMFs were made by matching them with the closest known modal frequencies (see Tables 2 and 3). The inclusion of other acceleration channels may enable the detection of longitudinal and transverse modes.

There are more peaks in the PDF from the ridesourcing data than there are in those from the controlled data. Furthermore, many of the peaks in the ridesourcing PDF appear notable, whereas in the other PDFs, there were 2–3 primary peaks and the others were negligible. This may suggest that there is a higher potential for false positives when dealing with uncontrolled datasets. On the other hand, this may be an artifact of a relatively small sample size that would resolve with larger volumes of data.

### Number of vehicle trips versus accuracy

**Fig. 4:**
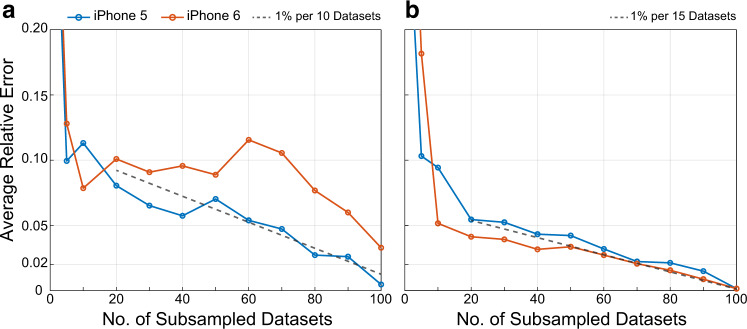
Errors of the most probable modal frequencies (MPMFs) as a function of the size of the data subset. **a** Smartphone MPMF errors for the first vertical frequency (*m* = 1). **b** Smartphone MPMF errors for the second vertical frequency (*m* = 2). Each line represents the mean error for a different smartphone.

The quantity of bridge trips in both tests is arbitrary: one could reasonably ask *"How many datasets are needed to accurately estimate a bridge modal frequency?”*. Answers to questions such as this could help produce practical guidelines that drive large-scale efforts to regularly collect vehicle scanning data. This was considered using random subsets of the controlled trips, i.e., choosing *N*_*S*_ < 102 bridge trips and repeating the analysis to extract MPMFs. The following analysis focuses on the first two modal frequencies as those were detected by both sensors previously. The average detection error (i.e. difference between MPMFs and true values) is provided in Fig. 4. Overall, these curves show that the frequency extraction procedure excels as more data becomes available.

Figure 4 a shows the behavior of the first frequency errors as data subsets grow larger and Fig. 4b shows the same relationship for the second frequency. In all cases, the MPMF errors fall to the order of 10% when the number of datasets reaches 10. Beyond this point, a gradual (not purely monotonic) increase in accuracy continues as the dataset size increases towards 100. With only 40 datasets, one can see that the iPhone 5 estimates for both frequencies become quite accurate; within 6% of the true values, which is also true for both smartphone estimates of the second modal frequency.

Overall, errors for the second frequency decreased more consistently and rapidly than those for the first frequency. In other words, less effort was required to estimate the second modal frequency with a high accuracy. With either smartphone, an estimate of the second modal frequency can be achieved within 5% of the true value with as few as 30 datasets; and with 50 datasets, the error reduces to 4%.

These plots indicate that, depending on the smartphone sensor, only a relatively small amount of datasets is needed to get a rough estimate of a modal frequency—between 10 and 50 datasets can achieve an error on the order of 10%; however, a considerably larger amount of data, about 80 or 90 datasets, is needed in order to reduce errors to the order of 3%. More specifically, once the error falls below 10% error, each additional 10 datasets tends to reduce it further by about 1%.

### Application on short highway bridge

The Golden Gate Bridge is an atypical bridge. Suspension bridges comprise less than 1% of US bridges. They have flexible main spans that usually exceed 500 m in length, corresponding to low fundamental modal frequencies (on the order of tenths of Hz). The proposed method was applied to a 28-m-long reinforced concrete bridge to evaluate its performance on a short span, which better represents a typical highway bridge. According to the Federal Highway Administration national bridge inventory, over 25% of US bridges have maximum spans between 15 and 50 m^53^. It is difficult to establish expected values for the fundamental frequencies of such bridges; however, they can be considered greater than 1 Hz^54,55^ and are therefore more likely to coincide with vehicle modal frequencies, which usually range 1–3 Hz^56,57^.

**Fig. 5:**
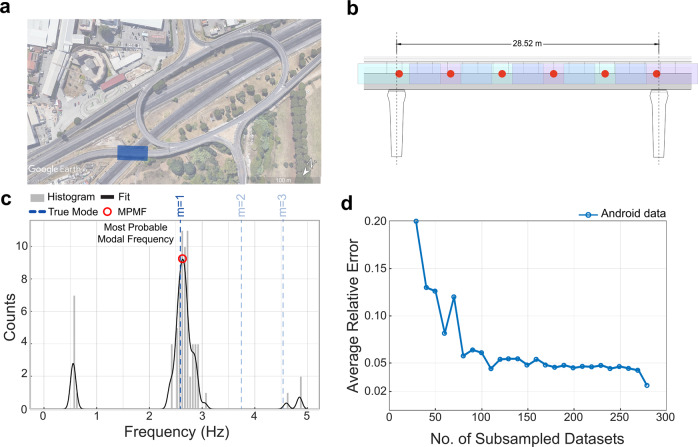
Crowdsensing application to a short-span concrete bridge. **a** An aerial view of the E80 highway exchange in Ciampino, Italy (image from Google Earth) with the studied bridge span highlighted in blue. **b** The geometry and spatial segmentation of the bridge. **c** The histogram and the fitted probability density function found from processing 280 smartphone datasets. The probability density function indicates a large peak at 2.63 Hz which is near the bridge’s fundamental frequency (*m* = 1) and is the only most probable modal frequency (MPMF). The reference frequencies for the first three modes are shown with blue vertical dotted lines. **d** The average error per dataset. The error reduces substantially as the number of scans increases. Overall, the error reduces more rapidly for 0 < *N* < 100 than for *N* > 100.

**Fig. 6:**
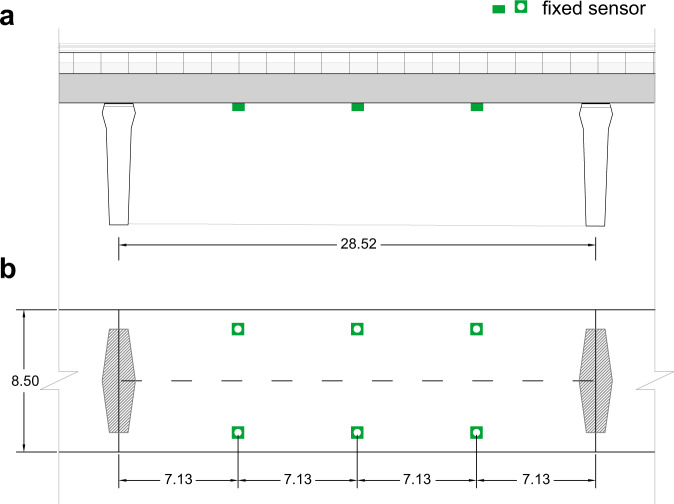
Network of fixed accelerometers on a short-span concrete bridge. The bridge is located at a highway exchange in Ciampino, Italy. All dimensions in meters.

The studied bridge is in Ciampino, Italy and is part of the European route E80 in the Rome metropolitan area monitored by ANAS Sp.A. The highway exchange is a system of ramps and turns, consisting of 30 short bridge spans (see Fig. 5a). The bridge span was instrumented with six accelerometers to record ambient vibrations nearly continuously from September 2020 to April 2021 (see Fig. 6 in “Methods” for the sensor layout). Successive batches of data (collected in two-hour intervals) were processed using the automated frequency domain decomposition (AFDD) algorithm^58^ to determine the structural modal properties of the bridge and track them over time (hours, days, weeks). Based on these analyses, the fundamental frequency (*m* = 1) of the bridge was determined as 2.58 Hz; this is the reference value for the mobile sensing results.

Smartphone-vehicle trips were recorded using an Android-based smartphone application that was developed by the researchers for the ANAS Sp.A. road maintenance crew; as such the data was partially controlled by the analysts (see Table 6 in “Methods”). The app was equipped with a geofence to automatically trigger data collection whenever the crew traveled over the bridge span of interest. Over 250 bridge trips were collected by the crew using mobile devices with the smartphone app. The proposed method processed 280 datasets to estimate MPMFs and compare with the identified fundamental frequency. Figure 5b–d illustrates the spatial segmentation pattern, the PDF of the frequency candidates using Gaussian KDE, and the error versus number of trips. As shown in Fig. 5c, the MPMF from mobile-sensor data matches the reference frequency (1.94% error). The PDF also shows some small, statistically insignificant peaks at other frequencies. The second and third frequencies were not detected in this batch of data. As observed when comparing controlled and ridesourcing datasets, the observability of modes depends on many physical factors such as bridge excitation, vehicle attributes, etc. Note vehicle-bridge-road interaction effects are not accounted for in the proposed method. Recent work has shown how information about the vehicle system, e.g., transfer function, and road profile can improve the accuracy of the estimated bridge modal properties^59–62^ (see Supplementary Note 4 for discussion).

**Table 6:** Definition of data controllability and comparison: What level of influence did the analysts have on the following data collection parameters? (*✓*= "Complete Control", ✕ = "No Control",  = "Guidance Provided, No Enforcement").

Parameter	Experiment	Ridesourcing	Highway Bridge
	(Controlled)	(Uncontrolled)	(Partially Controlled)
Smartphone Model	✓	✕	✕
Smartphone Mount Type	✓	✕	
Smartphone Orientation	✓	✕	
Smartphone Sampling Rate	✓	✕	✓
Vehicle Make and Model	✓	✕	✕
Vehicle Speed	✓	✕	
Vehicle Route	✓	✕	✕

Figure 5d, presents the effect of sample size on the fundamental frequency estimation error. The analysis conducted was identical to that which produced Fig. 4 (based on random subsets of data). As the number of samples increases to 100, the error drops to 5%. Beyond this point, the error continues to decline, at a much slower rate.

There are three main observations: First, similar to the error trends seen for the long-span bridge in Fig. 4, in this application, the errors decrease in two phases, a rapid change followed by a gradual one. The difference being the approximate *N* value at which this transition occurs (*N* ≈ 20 vs. *N* ≈ 100). Second, the error of the fundamental frequency estimate at *N* = 100 datasets is 5.5%, which is comparatively higher than the primary application (see Fig. 4). Part of this difference may be attributed to the uncontrollability of the data or the fact that trips on short-span bridges will generally contain fewer data samples due to short-time durations. Future work may utilize metadata on the uncontrolled factors such as smartphone mount, or vehicle model, to quantify estimation uncertainty. In addition, specialized methods for sensor noise reduction may be useful^37^. Finally, the spatial data aggregation technique was effective despite relatively imprecise GPS data. Reported GPS errors were about 4.3 m on average, which represented about 15% of the length of the bridge (see “Methods” for details). Analyses of noisy acceleration data in Supplementary Note 3 show similar results. Overall, the successful applications suggest that the proposed method may apply to a broad range of sensor qualities and bridge types.

### Discussion

This paper proves that bridge vibration frequencies can be identified from smartphone-vehicle trip data in real-world conditions. It is emphasized that data from a single trip is insufficient; yet as few as 100 crowdsourced datasets can produce useful modal frequency estimates (below 6 % error) for both short-span and long-span bridges. Collectively, the analyses of controlled and ridesourcing data (*N* = 174 total) produced accurate estimates of ten (seven unique) modal frequencies of the Golden Gate Bridge; five of which had an error of 0.000%. The number of trips considered in the primary study was less than 0.1% of the daily trips made on the Golden Gate Bridge; this shows that there is an enormous sensing potential represented by smartphones globally that contains valuable information about bridges and other important infrastructure. Furthermore, the accuracy of the most-probable modal frequencies (MPMFs) improved as the number of datasets increased.

Through a broad set of successful real-world applications, this paper strongly demonstrates the robustness of crowdsourced smartphone data for bridge health monitoring. The results exemplified three broad classes of crowdsourced data from which modal properties can be accurately extracted: controlled, uncontrolled, and partially controlled. This shows that analysts do not necessarily need to design or influence data collection features such as vehicle speed, smartphone orientation, etc., in order for the datasets to provide value to a bridge management system. Pre-existing mobile-sensor datasets, originally captured for other purposes, e.g., ridesourcing, public works, etc., may be repurposed for infrastructure monitoring.

The MPMF analyses did not explicitly account for vehicle-bridge-road-interaction effects yet were still successful in identifying bridge modal frequencies accurately. This highlights the strong and persistent traces of bridge modal frequencies among the collected datasets in varied vehicle and road conditions. Future applications that are more sensitive to vehicle-bridge-road-interaction features may benefit from incorporating smartphone metadata, e.g., smartphone mount, vehicle model, roadway type, etc., and vehicle dynamical properties to achieve precise MPMFs or to quantify uncertainties with regard to data controllability. In short, entropy is a desirable feature of crowdsourced vehicle trip data which will help reduce bias in estimated bridge properties (see Supplementary Note 3 for a detailed discussion). Recommended future work can quantify relationships between data controllability parameters and the accuracy of modal property estimates.

Long-term studies on bridge dynamic features over various environmental and operational conditions establish a foundation for the rapid development and verification of new methods for data-driven condition evaluations. Frequencies are a gateway to additional modal properties and structural features which have explicit relationships with certain types of bridge damage and deterioration, e.g., mode shape curvature, local stiffness, etc.^19,20,46,47,59,63^. Historically, such techniques have been confined to academic research applications, which have only impacted a small fraction of existing bridges^20,64–70^. Furthermore, the majority of these studies are based on short-term data (a few months); long-term structural behavior (at least one year of data) is essential to establish the baselines and performance benchmarks needed for condition evaluations^71^.

It is important to emphasize that machine intelligence, e.g., a trained AI model, was not needed to determine accurate MPMF results from aggregated smartphone-vehicle trip data. The real power of crowdsourced data lies in the ease of obtaining longitudinal data, which fuels a nearly continuous monitoring, feature extraction, and detection system with which a present state can be analyzed and compared to historical baselines (past seasons over years). With these unprecedented data volumes, automated structural condition assessment systems would no longer be confined to methods rooted in surrogate model optimization^72–74^; they could finally, truly benefit from advances in artificial intelligence and computational learning as seen in fields such as image and video recognition, recommendation systems, or multimodal sentiment analysis^75–80^. When fueled with long-term monitoring data, artificial intelligence has the potential to provide bridge engineers and owners with unprecedented information for maintenance and operation at virtually little to no extra cost.

The potential benefits of this paradigm are quantified in analyses (see Supplementary Note 5) which generate reliability profiles to simulate how bridge service life is affected by the availability of condition information in a maintenance plan. The analyses predicted that crowdsourced monitoring accumulates information that adds over two years of service to a 43-year-old bridge (15% increase) and adds almost fifteen years of service to a brand new bridge (30% increase). The findings of this paper could have an immediate impact on the management of bridges in many countries; they emphasize the benefit of integrating crowdsourced monitoring information into a bridge management plan as soon as the bridge is in operation.

## Methods

### Data controllability definitions

The data considered in this paper represent three broad classes of crowdsourced data: controlled, uncontrolled, and partially controlled. Seven controllability dimensions were considered to characterize the smartphone-vehicle trip data: smartphone model, smartphone mount type, smartphone orientation, smartphone sampling rate, vehicle make and model, vehicle speed, and vehicle route. In its simplest terms, controllability metrics address the following question: What level of influence did the analysts have on the following data collection parameters? For each dimension, there are three possible categories: Complete Control, No Control, and Guidance Provided, No Enforcement. Complete control means the analysts dictated this dimension, while No Control means the data collector controlled this dimension. Guidance Provided, No Enforcement means the analysts provided a formal recommendation to the data collector; however, the recommendation was not monitored or enforced whatsoever.

Controlled data is defined as a dataset for which analysts had complete control over all seven controllability dimensions. Uncontrolled data is defined as a dataset in which analysts had no control over any of the seven controllability dimensions. Partially controlled data are data that are neither controlled nor uncontrolled. Table 6 provides details on the controllability of each dataset.

In summary, the experimental data collected on the Golden Gate Bridge was controlled, the ridesourcing data collected on the Golden Gate Bridge was uncontrolled, and the data collected on the highway bridge in Italy was partially controlled.

### Controlled Data

The controlled data were recorded by an iPhone 5 and iPhone 6 using the Sensor Play App which recorded about one dozen data fields including sensor measurements, e.g., triaxial acceleration, GPS, gyroscope, magnetometer, etc., and inferred values, e.g., speed, GPS error. The trip data was collected in morning and afternoon rush-hour periods in five consecutive days (June 18–22, 2017). In the raw data, triaxial acceleration was sampled at about 100 Hz and GPS coordinates were sampled at about 1 Hz. The acceleration data was irregularly sampled. Temporal jitter was observed in most of the individual samples, i.e., non-uniform sampling periods. Additionally, when comparing sampling rates of independent datasets (different trips), the average actual sampling rate varied about 1–5% from the target value (the data was often undersampled). Therefore each acceleration dataset was resampled to 100 Hz before processing. Since the positions and orientations of the sensors were known, the acceleration channel used in the analysis was the one which coincided with gravity. In summary, the measurement channels used to determine MPMFs were triaxial acceleration and GPS.

Vehicle trips in the controlled dataset were completed during morning and afternoon rush hour periods. For each trip, the driver and passenger qualitatively rated the traffic levels on a 1−3 scale, where 1 means no congestion and 3 means stop-and-go conditions. Overall, these ratings were not significant, and only helped to roughly correlate traffic with vehicle speed. Nonetheless, it is important to track traffic volumes, e.g., car counts, as it directly influences the amplitudes of the bridge vibrations.

As a form of controlled variety in the trips, two sedan-style vehicles were used and five target speeds were defined: 32, 40, 48, 56, and 64  km h^−1^ (the speed limit on the bridge is 72 km h^−1^; however, there are 40 km h^−1^ advisories for areas near the toll gates, located at the south end of the bridge). The vehicle speed was monitored visually by the driver (cruise control was not used). Instantaneous speed data (automatically produced through the app) was used to compare with the speed targets. Overall, the median speed values matched well with the target speeds. The first fifty trips were completed using a Nissan Sentra and the remaining fifty-two trips used a Ford Focus. Each vehicle trip was assigned a predetermined target speed; while crossing over the bridge spans, the driver almost always (during one northbound trip, the driver was ordered by an authority to increase the speed of the vehicle; this resulted in an average speed well above the target speed of 48 km h^−1^. During one southbound trip, high traffic congestion resulted in an average speed that was well below the target speed of 32 km h^−1^. Two extra trips were made to account for these instances, hence 102 in total) maintained an average speed within ± 4 km h^−1^ of the target. A summary of vehicle trip details, e.g., the trip speed distribution is provided in Table 7.

**Table 7:** Details of the 102 vehicle trips made over the Golden Gate Bridge.

Target avg. speed	No. of trips	Avg. traffic rating
32	26	1.5
40	29	1.2
48	17	1.2
56	20	1.1
64	10	1.0

Trips were completed using two different vehicles driving with five constant speeds. The distribution of the trips taken for each speed is provided. The majority of the trips were at 40  km  h^−1^ (29 out of 102) and the minority of the trips were at 64 km h^−1^ (10 out of 102). Traffic ratings were recorded qualitatively on a scale of 1-3 for each trip based on observations of the researchers. A rating of 3 indicates very high traffic, e.g.,bumper-to-bumper.

### Uncontrolled ridesourcing data

Vehicle trips supplied by the ridesourcing operator, Uber, were made by 37 distinct vehicle types that together constitute a set of typical vehicle makes and models used in such fleets. The largest group, 9 trips were made by Toyota Priuses; a breakdown of the number of trips by vehicle type is presented in Table 4. Measurements were collected by the ridesourcing app itself during regular operations in its default commercial-use settings, i.e., no modifications were made to accommodate bridge vibration analysis. The analysts had no control over the data prior to its collection, i.e., no control over parameters listed in Table 6. The ridesourcing operator, Uber, provided data as requested, exclusively based on a geographical area of interest (the Golden Gate Bridge), without any other specifications.

Each dataset (trip) contained about one dozen data fields, which included sensor measurements, e.g., triaxial acceleration, GPS, gyroscope, magnetometer, etc., inferred values, e.g., speed, GPS error, and vehicle and smartphone model information. The triaxial acceleration data was provided in a raw, unknown orientation, which required correction in order to extract the channel corresponding to the vertical (gravity) direction. The Nericell approach^81^ was selected to reorient the acceleration signals. This method uses triaxial accelerations and GPS to estimate tilting angles and reorients them based on Euler angles. The average GPS error value was about 7.7 m. In summary, the analysis to determine MPMFs used only triaxial acceleration and GPS data.

**Fig. 7:**
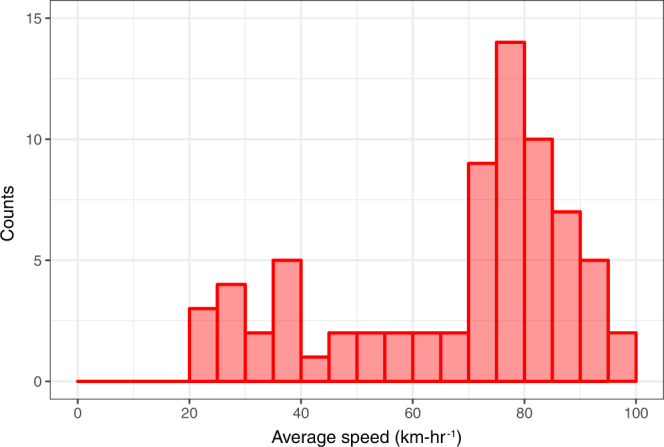
Distribution of vehicle speeds. The average speed was calculated for each trip in the ridesourcing dataset. This figure plots a histogram of the average vehicle speeds (bin size is 5 km-hr^−1^).

Drivers used multiple types of smartphones, a summary of which is given in Table 5. In total, in 19 trips, the device used was an iPhone model, ranging from iPhone 5s to iPhone X and including multiple model variants. In 51 trips, the device was a Samsung model, with the Galaxy S5 and Note 4 being the most popular (17 and 10 trips respectively). The vehicle speed histogram in Fig. 7 shows that most trips were driven at relatively high speeds, above 70 km h^−1^ (43 mile.hr^-1^), while the rest of trip speeds were distributed relatively evenly below that; it was assumed that this resulted from varying traffic conditions on the bridge.

### Partially controlled data

For the short-span highway bridge, 280 smartphone-vehicle trips were recorded using an Android-based smartphone application that was developed by the research team and was used by the ANAS Sp.A. road maintenance crew. The app is equipped with a geofence to automatically trigger the data collection whenever the crew traveled over the bridge of interest. The data collected included direct measurements of triaxial acceleration, GPS, gyroscope, and inferred values such as orientations (rotation) and GPS error. The rotation vector is a post-processed vector produced by Android that represents the location and orientation of the device. In summary, the provided rotation vector consists of the last three components of the unit quaternion that comprise the necessary information for device reorientation. A comprehensive description of the reorientation method using quaternion vectors is presented in^82,83^. The Nericell reorientation approach used for the ridesourcing data was not suitable because orientations were already available through the data collection app. After reorienting the triaxial acceleration signals, the channel corresponding to gravity was selected for further processing.

**Fig. 8:**
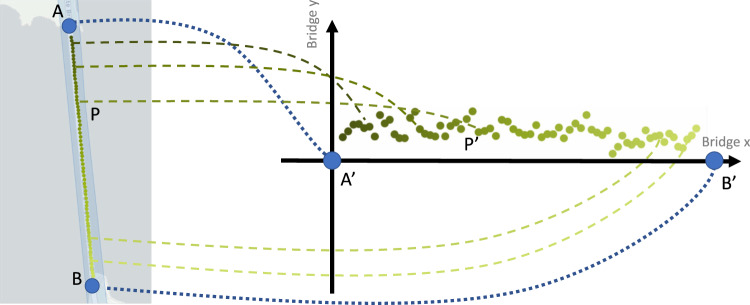
Illustration of transforming GPS points to bridge coordinates. The vehicle (mobile sensor) collects samples as it travels on the bridge from lat-long points *A* to *B* (left panel). The gradient of the points indicates the temporal dimension: the point with the lightest color is the most recent. On the right panel, the GPS points are mapped from lat-long values to a bridge coordinate system where $A^{\prime}$ and $B^{\prime}$ represent points at each end of the bridge and correspond to GPS points *A* and *B*, respectively. Note that in the real application, the location of the towers were used as reference points *A* and *B*, since our analysis only considers the main span of the bridge that has a total length of 1280 m.

It is important to note that the Nericell approach relies on GPS measurements which generally have lower SNRs on shorter bridges. The GPS sampling rate for the smartphone used in this research is 1 Hz. The average GPS accuracy reported by the operating system of the device was 4.3 m with regard to a 28-meter-long bridge. The Uber dataset showed noisier GPS data with an average error of 7.7 m, yet the bridge considered was 1280 m long. To preprocess the GPS data, the coordinates are first projected to a 1D axis along with the longitudinal axis of the bridge (see Fig. 8). Next, the data points with GPS coordinates outside predefined bounding boxes were discarded. After removing outliers, the missing coordinates were recalculated by linear interpolation. The GPS errors did not appear to negatively impact MPMF estimation. Overall, the spatial segmentation and aggregation approach (especially with overlaps) helps to mitigate inaccurate spatial coordinates caused by GPS errors. Future work attempting to extract spatial information of bridge vibration, e.g., structural mode shapes, may require additional processing such as advanced GPS filtering, clustering, and/or sensor data fusion for improved location estimation, e.g. simultaneous localization and mapping^84^.

In total, nine different phone models were used and vehicle speeds varied from 9 to 72 km h^−1^ (median of 46.8 km h^−1^). More than one vehicle was used for data collection; however, the precise number of different automobiles included was not recorded and is unknown to the analysts. The drivers crossed the bridge during their regular commutes; they were not provided any guidance on vehicle routes, smartphone models, vehicle types, or trip quotas. The analysts recommended drivers to drive at constant speeds and to mount their phones on a solid holder. The analysts had no control over the smartphone model or vehicles used for data collection and this metadata was not provided for each trip.

### Space-frequency representation of the signal

The instantaneous vertical acceleration measurement at location *r* on the bridge is represented as a linear combination of oscillatory modes plus noise:

1 $$x(r,t)=\mathop{\sum }\limits_{d=1}^{D}{A}_{d}(t){{{\Phi }}}_{d}(r)\cos (2\pi {\omega }_{d}t)+e(t)$$

where *A*_*d*_(*t*) is a time-dependent amplitude, Φ_*d*_(*r*) describes the spatial mode shape and *ω*_*d*_ is the vibrational frequency of mode *d*, while *e*(*t*) represents the noise. Our main goal is to identify the *ω*_*d*_ frequencies given the signals *x*_*i*_(*t*) ≡ *x*(*r*_*i*_(*t*), *t*),  *i* = 1, 2…102, where the correspondence between bridge coordinates and time, i.e. the *r*_*i*_(*t*) function is given empirically based on the GPS measurements for each bridge crossing in our dataset. Mathematical connection between these entities and bridge vibrations is discussed in more detail in the Supplementary Note 1. To achieve this, the time-frequency representation of signal *x*_*i*_(*t*) is analyzed based on the assumption that the effective amplitudes, ${A}_{di}^{{{{{{{{\rm{eff}}}}}}}}}\equiv {A}_{d}(t){{{\Phi }}}_{d}({r}_{i}(t))$ are slowly varying functions of time. This is essential since contributions of different modes in the signal vary systematically and stochastically based on the mode shapes and the dynamic forces acting on the bridge; along with the presence of noise, this makes simple spectral methods, e.g. those based on the Fourier analysis of the whole signal unsuitable. The synchrosqueezed wavelet transform^48,85,86^ was employed based on its effectiveness at recovering instantaneous frequencies of noisy signals with many harmonic-like components. This way, for signal *x*_*i*_(*t*), a complex valued function ${T}_{{x}_{i}}(\,f,t)$, is obtained, which gives the instantaneous amplitude of the modal frequency *f* at time *t*. Then, the time variable is replaced by its corresponding location *r*_*i*_(*t*) in bridge coordinates for each trip in the dataset, obtaining ${T}_{{x}_{i}}(\,f,r)$, the space-frequency representation of our signal. While in theory, *r* is often considered a continuous variable, in the following, a discretized version is used which corresponds to a signal downsampled to 1 Hz in time.

### Spatial analysis of bridge vibrations

Modal analysis describes how the presence of each structural vibration mode varies over space and time (see Supplementary Note 1 for detailed formulation). The synchrosqueezed wavelet transform constructs time-frequency representations to quantify how frequency content in the mobile-sensor data varies over time. In mobile acceleration data, the recorded signal includes a mixture of the spatial vibrations of the bridge. The spatial analysis approach in this paper consists of two steps: (i) transformation of GPS points to bridge coordinates; and (ii) spatial segmentation of the bridge. This section describes the first step; the following section describes the second step.

Figure 8 illustrates the first step of the process in which the GPS data are mapped to points on the bridge. For this, it is helpful to use a digital map to select points at the corners of roadway of the bridge which act as a frame of reference; these points should coincide with the start and end of the bridge. With these reference points established, the haversine formula can be used to calculate distances between GPS points and transform them into another coordinate system. The bridge coordinate system is viewed as a 2D Cartesian space defined based on the reference points. Figure 8 shows how the reference points *A* and *B* on a map (satellite view) are mapped to points $A^{\prime}$ and $B^{\prime}$ in the bridge coordinate system. When possible, it is important to verify that the calculated distances, e.g., $\overline{A^{\prime} B^{\prime} }$, are consistent with the known geometry of the bridge. In addition, the quality of the GPS data should be inspected. Once the sensor positions are represented in the bridge coordinate system, the signal-to-noise ratio (SNR) of the points will vary for each dimension; generally, for smartphones, the SNR will be significantly higher in the primary traveling direction (parallel to the roadway, "Bridge *x*” in Fig. 8). In this introductory application, it is sufficient to use only one dimension, e.g., *x*-coordinates, when describing the locations on the bridge at which data is being collected. In further applications, it may be necessary to use both dimensions.

### Spatial segmentation of a bridge

A fixed number of discrete, uniform overlapping segments are defined over the length of the bridge. The start and end of the spatial segmentation scheme are consistent with the reference GPS points chosen earlier to coincide with the bridge coordinate system. Figure 1 shows discrete segments along the length of the bridge which have two defining parameters: the segment length, *c*, and the distance between the centers of adjacent segments, Δ*s*. The overlap of two adjacent segments is then a function of *c* and Δ*s*, namely, *c*_*o*_ = *c* − Δ*s*. The bridge segments, *bars* = [*s*_1_, *s*_2_, . . . , *s*_*M*_], collectively define an interval in one-dimensional space, $[{s}_{1}-\frac{c}{2},{s}_{M}+\frac{c}{2}]$. Connecting these to the measurements, for the *i*th bridge crossing, there is a discrete set of *r*_*ij*_ points that fall inside each bridge segment.

When selecting these parameters, it is important to consider the vehicle speeds, sensor sampling rates, and the geometry of the bridge, among other factors. The spatial segmentation of the Golden Gate Bridge was set to have a large number of wide segments. A large number of segments (larger *M* and smaller Δ*s*) increases the number of total entries in the frequency candidates vector, ${\hat{f}}_{s}$ (see below), which improves the resolution of subsequent PDF estimates. Furthermore, wide segments (larger *c*) improve overall robustness to noise; if the frequency net in space is too small, then noisy, spontaneous frequencies, that are unrelated to the bridge’s vibrations, may appear to be statistically significant. For the primary application (controlled data), the spatial segmentation scheme was set to have segment centers separated by 10 meters, or 129 equally spaced segments (Δ*s* = *L*/129) and the width of each segment was set to 258 meters (*c* = *L*/5, 20%). For the ridesourcing data (uncontrolled), the width of each segment was *c* = 3*L*/20 (15%).

### Determination of the Most Probable Modal Frequencies (MPMFs)

The space-frequency representation of the signal (${{{{{{{{\bf{T}}}}}}}}}_{{x}_{i}}(\,f,r)$) is used as a basis of a statistical aggregation method that aims to select frequencies that are present consistently in the bridge vibrations and among the vehicle trips in our experiments. Note that a later extension of our method would easily allow to focus on frequencies that are present only in some locations, following the pattern of the associated spatial mode. The end result of our method is a probability distribution with peaks representing possible modal frequencies, from which the most probable modal frequencies (MPMFs) are identifiable.

The first step is to identify the local maxima within $| {{{{{{{{\bf{T}}}}}}}}}_{{x}_{i}}(\,f,r)|$, which are related to the ridges of the synchrosqueezed wavelet transform (see the following section for further details on this process).

Start with a set containing all local maxima of $| {{{{{{{{\bf{T}}}}}}}}}_{{x}_{i}}|$ for each discrete location *r*_*ij*_ for each bridge crossing. Only statistically significant peaks from each set will be kept, i.e., those with a prominence in the top *α* percentile of the corresponding empirical cumulative distribution function (CDF). The result is a family of piece-wise ridges for each dataset, as illustrated in Fig. 2a. The process is repeated for all *N* datasets. Then the ridges inside each bridge segment, ${s}_{m}\in \bar{{{{{{{{\bf{s}}}}}}}}}$ are aggregated for each bridge crossing.

Next, with a shared bridge segmentation scheme, the prominence values of all statistically significant ridges are aggregated among the whole dataset as well. In other words, all datasets are aggregated (see Fig. 2) to produce a matrix of the cumulative prominence values, **P**_*N*_, with respect to all frequencies, *f*, and all segments, $\bar{{{{{{{{\bf{s}}}}}}}}}$. Then, for each ${s}_{m}\in \bar{{{{{{{{\bf{s}}}}}}}}}$, the prominence values are sorted in descending order, and the frequencies associated with the top *N*_*R*_ values are stored in a vector ${\hat{{{{{{{{\bf{f}}}}}}}}}}_{{s}_{m}}$, which is assigned to *s*_*m*_. These vectors are combined to create the frequency candidate vector ${\hat{{{{{{{{\bf{f}}}}}}}}}}_{s}$, which has *M* × *N*_*R*_ entries.

The final probability distribution function (PDF) (shown in Fig. 2c as a histogram) is produced using the frequency candidate vector. This empirical PDF is expected to be multimodal, with each mode representing a possible vibrational frequency. The MPMFs can be defined as a subset of the possible modal frequencies having the largest amplitudes. At this stage, the problem of determining the MPMFs, relies on the method used for estimating the PDF, as the MPMFs are local maxima in the PDF. A kernel density estimation is used to fit a smooth, nearly continuous PDF; this improves the precision with which MPMFs are estimated.

The procedure for computing the MPMFs for *N* datasets is summarized below. In addition to defining bridge segmentation parameters, it is necessary to select parameters *α* and *N*_*R*_ which impact ridge estimation and spatial frequency aggregation. In practice, *α* ≤ 0.05 and *N*_*R*_ = 5 were used.

1. Vertical acceleration signal *x*_*i*_(*t*) and linear position in bridge coordinates *r*_*i*_(*t*) is extracted from the measurements for *i* = 1, 2,…*N* datasets.
2. Acceleration signal is filtered and downsampled to the desired frequency *f*_cut_. Time and space are discretized accordingly: *t*_*ij*_ = *j*/*f*_cut_ and *r*_*ij*_ = *r*_*i*_(*t*_*ij*_) with *j* = 1, 2…*M*_*i*_. Note that time and space discretization along with the value of *M*_*j*_ can differ among the measurements in the dataset if vehicles travel at different speed. Note that this is not an issue because a common spatial segmentation of the bridge is implemented later. The Golden Gate Bridge analyses used *f*_cut_ = 0.5 Hz.
3. Synchrosqueezed wavelet transform is calculated for each measurement separately, resulting in the ${T}_{{x}_{i}}(\,f,{t}_{ij})$ time-frequency representation of the signals. Morlet wavelets were employed in this study.
4. The frequency-time pairs are mapped to frequency-space pairs, i.e., (*f*, *t*_*ij*_) → (*f*, *r*_*ij*_). This change of variables remaps the synchrosqueezed wavelet transform accordingly ${T}_{{x}_{i}}(\,f,{t}_{ij})\to {T}_{{x}_{i}}(\,f,{r}_{ij})$.
5. Define _*Pi*_(*f*, *r*_*ij*_) as the local maxima of $| {T}_{{x}_{i}}(\,f,{r}_{ij})|$ ∀ *i*, *j*
6. Select a value for *α*. Compute ${F}_{{P}_{i}}(\,f| {r}_{ij})$, the empirical CDF of ${P}_{{x}_{i}}(\,f,{r}_{ij})$ ∀ *i*, *j*. Define the statistical ridges as the values in the upper *α*-percentile: _*Pi*_(*f*_1−*α*_, *r*_*ij*_) where *f*_1−*α*_ are the values associated with ${F}_{{P}_{i}}(\,{f}_{1-\alpha }| {r}_{ij}) > 1-\alpha$
7. Aggregate the local frequency maxima using the summation ${P}_{N}(\,f,{s}_{m})=\mathop{\sum }\nolimits_{i = 1}^{N}{\sum }_{{r}_{ij}\in [{s}_{m}-\frac{c}{2},{s}_{m}+\frac{c}{2})}{P}_{{x}_{i}}(\,{f}_{1-\alpha },{r}_{ij})$
8. Select a value for *N*_*R*_. Define ${\hat{{{{{{{{\bf{f}}}}}}}}}}_{{s}_{m}}$ as the extracted frequency vector which contains the frequencies corresponding to the *N*_*R*_ largest values in *P*_*N*_(*f*, *s*_*m*_). Combine all *M* vectors to construct the frequency candidate vector ${\hat{{{{{{{{\bf{f}}}}}}}}}}_{s}=[{\hat{f}}_{{s}_{1}},{\hat{f}}_{{s}_{2}},...,{\hat{f}}_{{s}_{M}}]$ (this constitutes aggregation in space).
9. Finally, estimate the PDF of ${\hat{{{{{{{{\bf{f}}}}}}}}}}_{s}$ using kernel density estimation, from which the most probable modal frequencies (MPMFs) are determined. For reference, in this study Gaussian kernels were used with a bandwidth equal to 1% of the frequency range.

### Piecewise wavelet ridges

A ridge is a sequence of frequencies, **f**(*r*), that follow a curve and trace maximum amplitudes^87–90^. Ridge extraction methods usually consider either the transform’s modulus or its instantaneous phase. In applications with noisy data, modulus-based methods are often preferred^91,92^. Ridge extraction methods generally look for a smooth function (or set of functions), *r* → ∣**T**_*x*_(**f**_*R*_(*r*), *r*)∣, that concentrates most of the energy in the time-frequency domain. The nature of the estimated ridges rely on the complexities of the underlying signal components as well as the extraction method. For example, if a signal has only one component, *x*_1_(*t*) = **A**_1_(*t*)*cos*(2*πφ*_1_(*t*)), its true ridge is unique and equivalent to the instantaneous frequency function, ${{{{{{{{\bf{f}}}}}}}}}_{R}(t)=\varphi ^{\prime} (t)$^86^.

In these applications, the signal has numerous underlying components, which have distinct characteristics that influence ridge extraction: (i) the components have time-invariant frequencies (a direct result of Eq. (S2) in Supplementary Note 1); (ii) the components’ amplitudes are intermittent; they are, by nature, stochastic and can vary quickly in time as they rely on an unknown dynamic excitation (*p*(*t*) of Eq. (S1) in the Supplementary Note 1); (iii) as a corollary, some components are expected to be present in one dataset, but absent in another. These characteristics call for a multiridge detection technique^93,94^ that is capable of rapid switching between components whenever one disappears or another emerges. The approach used here is motivated by the method developed by^95^ for speech analysis, which can accommodate rapid changes in spectral peaks, i.e., sudden births and deaths of components in time.

### Robustness analysis

To estimate the robustness of the methods, all analyses were repeated for random samples selected from the data. For *N*_*S*_ = 1, 5, 10, 20, 30, 40, 50, 60, 70, 80, and 90, *N*_*S*_ bridge trips were selected at random and analyses were repeated considering this random sample as an input. For each *N*_*S*_ value, random sampling and estimation are repeated 100 times; in each case, the top frequency is selected (mode of the histogram created from the corresponding ${\hat{{{{{{{{\bf{f}}}}}}}}}}_{s}$ values) and compared with the candidate true modal frequencies of the Golden Gate Bridge. The average error is calculated based on the closest candidate and also count the number of times the error is less then 5%.

Furthermore, the bridge-vehicle system is simulated and the signal is analyzed with the same methodology. This process yields similar results to what was observed in the real data, as shown in Supplementary Note 3 and Figs. S1 and S2.

### Supplementary information

Supplementary Information

## Data Availability

All figure source data and raw data for the "Controlled Data” analyzed in this paper have been uploaded to Dryad as "Source Data for Crowdsourcing Dynamic Bridge Monitoring with Smartphone Vehicle Trips" (10.5061/dryad.zs7h44jcw). Any access to the controlled data must be cited as shown in^96^. The uncontrolled ridesourcing data that support the findings of this study are available from Uber but restrictions apply to the availability of these data, which were used under license for the current study, and so are not publicly available. Data are however available from the authors upon reasonable request and with permission of Uber. The partially controlled data that support the findings of this study are available from ANAS Sp.A. but restrictions apply to the availability of these data, which were used under license for the current study, and so are not publicly available. Data are however available from the authors upon reasonable request and with permission of ANAS Sp.A.

## Code availability

Code to replicate this research can be requested from the corresponding author.
