# Supplementary material for: Crowdsourcing bridge dynamic monitoring with smartphone vehicle trips
Source: Commun Eng. 2022 Nov 3;1:29. doi: 10.1038/s44172-022-00025-4 (PMC10955948; doi:10.1038/s44172-022-00025-4)
Supplement: Supplementary file 1 — Supplementary Information [file 44172_2022_25_MOESM1_ESM.pdf]

Crowdsourcing Bridge Dynamic Monitoring with Smartphone Vehicle Trips
Supplementary Material

Thomas J. Matarazzo*, Dániel Kondor, Sebastiano Milardo, Soheil S. Eshkevari
Paolo Santi, Shamim N. Pakzad, Markus J. Buehler, and Carlo Ratti
* E-mail: `tomjmat@mit.edu`

Supplementary Note 1: Brief Review of Bridge Dynamics

Bridges are structural systems that respond dynamically to excitation forces, e.g., traffic, wind, etc. in accordance with the wave equation. In particular, the equation of motion defines this relationship explicitly in terms of the structure's physical properties

$$\sum_{j=1}^{N} m_{ij}\ddot{u}_j(t) + \sum_{j=1}^{N} c_{ij}\dot{u}_j(t) + \sum_{j=1}^{N} k_{ij}u_j(t) = p_i(t) \qquad \text{(S1)}$$

where $\mathbf{m}$, $\mathbf{c}$, and $\mathbf{k}$ are the discrete-space mass, damping, and stiffness matrices and $\ddot{u}_i(t)$, $\dot{u}_i(t)$, $u_i(t)$ (with $i = 1, 2 \ldots N$) are the accelerations, velocities, and displacements of the structure at N specified degrees of freedom, and the dynamic forces are given by $p_i(t)$. In summary, $\mathbf{m}$, $\mathbf{c}$, and $\mathbf{k}$ are $N \times N$ matrices and $\mathbf{u}(t)$, $\dot{\mathbf{u}}(t)$, $\ddot{\mathbf{u}}(t)$, and $\mathbf{p}(t)$ are $N \times 1$ vectors. The current work focuses on *vertical* displacements and the components are indexed accordingly using subscript i, i.e. $u_i(t)$ describes the vertical displacement of the bridge at location $r \equiv \delta i$ (where δ is the spatial discretization used in the model). In this study, a very large number of degrees of freedom, N, is considered to attain a very fine discretization in space, as this is consistent with the measurements of a mobile sensor network.

For linear structural systems, the dynamic response can be represented as a summation of vibration modes [1] that are the solution of the homogeneous equation (i.e. the $p(t) = 0$ case). These can be written as $\mathbf{\Phi}_d q_d(t)$ for $d = 1, 2, \ldots N$, where $q_d(t) = \Re e^{i\Omega_d t}$ are scalar harmonic functions, and $\mathbf{\Phi}_d$ are $N \times 1$ vectors obtained as a solution of the generalized eigenvalue equation $-\Omega_d^2 \mathbf{m}\mathbf{\Phi}_d + i\Omega_d \mathbf{c}\mathbf{\Phi}_d + \mathbf{k}\mathbf{\Phi}_d = 0$. The eigendecomposition results correspond to the modal properties of the structure: mode shapes are given by the $\mathbf{\Phi}_d$ vectors, while the eigenvalues $\Omega_d \equiv \omega_d + i\zeta_d$ give modal frequencies ω_d and damping rates ζ_d. The solutions of Eq. (S1) can then be represented as a linear combination of individual vibrational modes:

$$u_j(t) = \sum_{d=1}^{N} c_d(t)\Phi_{dj} q_d(t) \qquad \text{(S2)}$$

For a system described by N degrees of freedom, there can be up to N vibrational modes contributing to its movement at any time. In the homogeneous case, the c_d amplitudes are constants determined by the initial conditions, while in the general case with time-varying $\mathbf{p}(t)$ excitation, the $c_d(t)$ coefficients will be time dependent as well. Nevertheless, in many cases, the rate of change of the $c_d(t)$ amplitudes is slow compared to the time-frame determined by the corresponding ω_d modal frequency, thus treating the behavior of the bridge as a sum of vibrational modes with time-varying amplitudes is justified, with each mode having a different contribution to the response at different times and locations. The spatial component is often well modeled as a sinusoid as well, i.e. the jth component in $\mathbf{\Phi}_d$ is given by $\Phi_{dj} = \sin 2\pi\delta j/\lambda_d + \varphi_d$, where λ_d and φ_d are the wavelength and phase of mode d.

Note that this representation is consistent with that given in Eq. (1) in *Methods*. We gain the form in Eq. (1) by switching to the continuous variable r to represent space, adding the noise term $e(t)$ and incorporating damping (ζ_d) and the effect of continuous excitations from $p_i(t)$ in the combined amplitude term $A_d(t)$. The presence of a modal response in $u_j(t)$ is amplified when (i) the dynamic force has a spatial distribution that is strongly correlated to the mode shape; and/or (ii) the dynamic force has spectral content that is very close to a modal frequency.

Theoretically, N terms are present in Eq. (S2). For efficient computation, modal truncation is often practiced, in which the summation includes only the first $\bar{N}$ terms where $\bar{N} \ll N$ and serves as an accurate approximation for $u(t)$. In other words, modal truncation assumes that the first $\bar{N}$ modes dominate the response. Note in practice, it is difficult to confirm linear structural behavior defined in Eq. (S1) because the internal and external dynamic forces are unmeasured and unknown. Suspension and stay-cable bridges, in particular, can exhibit strong geometric nonlinearities, especially in tower and cable dynamics. Regular bridge traffic is an "operational load" or an "ambient excitation" in the structural health monitoring community. Under "ambient vibration" conditions, i.e., as long as the excitation amplitudes remain relatively low, the bridge displacements are not large and the measured responses on the bridge deck are well characterized by the linear equation of motion [2–6]. This approach is used widely and is embedded within numerous benchmark approaches such as *ERA-NExT* [7] and *FDD* [8].

In summary, the physical response, e.g., $u_j(t)$, is two-dimensional: it depends on space and time. Modal analysis shows that the response to dynamic loads has a unique spatial-spectral composition based on intrinsic modal vibration properties. The underlying vibration modes can be observed by analyzing measurements of the physical response, i.e., sensor data.

Supplementary Note 2: Wavelet-Based Time-Frequency Representation of Recorded Signal

A primary objective of the analysis is to accurately determine the frequency content within the measured vehicle-bridge response. The structural response of the bridge is sampled in time and space through mobile sensing. The resulting signal may be non-stationary and non-linear, with frequency content that is highly dependent on time. Therefore, to avoid improper assumptions on the physical and statistical nature of the signal, a time-frequency representation is needed to evaluate the underlying frequency components. A plethora of time-frequency methods have been employed for various structural health monitoring (SHM) applications such as the Short-time Fourier Transform [9], Empirical Modal Decomposition [10,11], Wigner-Ville distribution [12], and the Wavelet Transform [13]

Synchrosqueezing is an algorithm applicable to time-frequency representations of signals with time-varying spectral characteristics. The technique was introduced for wavelet transforms by [14] and further mathematical details and applications have been discussed in [15,16]. In this framework, the time-series signal $x(t)$ is represented in the general form

$$x(t) = \sum_{d=1}^{D} x_d(t) + e(t) \tag{S3}$$

where each signal component $x_d(t) = A_d(t)cos(2\pi\varphi_d(t))$ is a "Fourier-like oscillatory mode" with a time-dependent amplitude $A_d(t)$, time-dependent frequency $\dot{\varphi}_d(t)$, and signal noise $e(t)$ [14,15]. The ultimate goal is to obtain the amplitude $A_d(t)$ of instantaneous frequency $\dot{\varphi}_d(t)$ for each d . It is important to note the definition of $x(t)$ in Eqn. (S3) is compatible with the modal analysis of $u_j(t)$ in Eqn. (S2) and the $x_i(t)$ form presented as Eqn. (1) in the main text. Most importantly, we assume that $\varphi_d(t) \equiv \omega_d t$ and that $A_d(t)$ is determined by the Φ_{dj} mode shape at the $r(t)$ location the vehicle is at time t.

Identifying the signal components then is achieved using a three-step process.

First, the time-frequency representation of the signal $x(t)$ is produced using the continuous wavelet transform

$$W_x(a,b) = \frac{1}{a}\int_{-\infty}^{\infty} x(t)\overline{\psi\left(\frac{t-b}{a}\right)}dt \tag{S4}$$

where $\overline{\psi(\xi)}$ is the complex conjugate of a selected mother wavelet, e.g., Morlet, a is the scale parameter ($a > 0$), and b is the time offset parameter. Discretization of the time scale, b, follows that of the original discrete-time variable of the signal, t: a series of K values separated by Δt.

Second, in the pursuit of an instantaneous frequency estimate, the time derivative of the continuous wavelet transform is used to produce the phase transformation $\omega_x(a,b)$

$$\omega_x(a,b) = \frac{-i}{W_x(a,b)}\frac{\partial W_x(a,b)}{\partial b} \tag{S5}$$

where $i^2 = -1$. The phase transform can provide the exact instantaneous frequency in the case where $\varphi_d(t)$ is constant. Overall, $\omega_x(a,b)$ is comparable to a "FM demodulated frequency" estimate at (a,b). Note the partial derivative with respect to b, $\partial_b W_x(a,b)$, is equivalent to the one with respect to t, $\partial_t W_x(a,b)$ [15]. Also note that ω_x is undefined when $|W_x| = 0$; in practice, because of noise and other artifacts, a threshold γ is used to mitigate instabilities by ignoring points where $|W_x| \leq \gamma$ [15].

Third, the time-scale plane is transformed to a time-frequency plane in a process called synchrosqueezing; in other words, the scale variable is reassigned to a frequency variable. This results in the Wavelet Synchrosqueezed transform of $x(t)$, obtained as:

$$T_x(f,b) = \int_{\{a:|W_x(a,b)|>\gamma\}} W_x(a,b)\delta(\omega_x(a,b)-f)a^{-1}da \tag{S6}$$

where f is a frequency variable and $\delta()$ is the Dirac delta function. At this stage, a, b and f are usually discretized for efficient digital computation and $\mathbf{T}_x$ is a matrix calculated from discrete summations based on equation (S6). The discrete frequencies are distributed logarithmically, defined as $\mathbf{f}_l = \frac{1}{K\Delta t}2^{l\Delta f}$ with index $l = 1, 2, \ldots n_a$, for a signal with K samples in time with sampling period Δt, for n_a desired discrete frequencies and where $\Delta f = \frac{1}{n_a-1}\log_2(K/2)$. For the sake of consistency among signals of different length, the frequency resolution is commonly defined by n_v, the number of "voices" in an octave, i.e. in an interval in which the frequency is doubled: $n_a = Ln_v + 1$, where $L = \log_2(M/2)$ is the number of frequency octaves possible in a signal of M samples [17].

Supplementary Note 3: Noise Sensitivity Analyses

Smartphones collect noisy inertial measurement unit data. In order to provide guidance for the hyperparameter selection and establish sensitivity to measurement noise, we conducted a numerical simulation of the bridge-vehicle system based on identified modal characteristics of the Golden Gate Bridge from the latest comprehensive monitoring project [18]. The bridge is modeled as a multi-degree-of-freedom linear system with similar geometries to the original structure. For operational, ambient vibrations, it is common practice to assume the structural system and material behave linearly. In order to take the vehicle-bridge interactions into account, a simplified modeling approach is adopted from Eshkevari et al. [19]. In summary, the approach posits that due to the negligible weight of a sensing vehicle compared to a long-span bridge, the dynamic analysis of vehicle-bridge interacting system can be decoupled. This in turn, dramatically reduces the computational costs and model complexity while maintaining the accuracy of estimations high.

In this numerical study, different signal-to-noise ratio (SNR) levels are simulated in order to investigate the effect on the accuracy of estimations as well the minimum required number of trips to reach a predefined estimation confidence threshold. To capture the effect of the random traffic load,

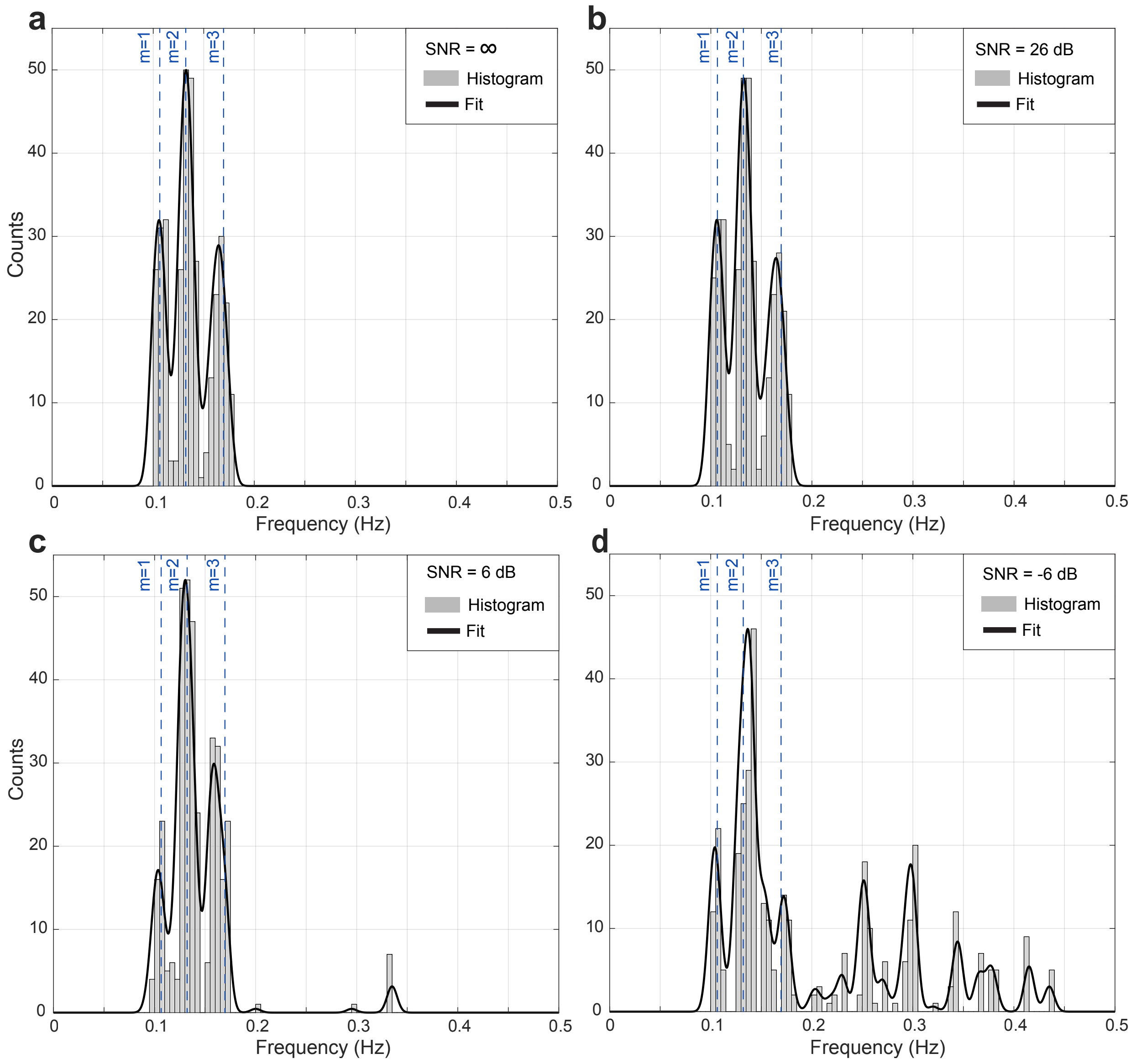

Figure S1: Histograms and estimates of probability density functions (PDFs) for simulated data with varyious signal-to-noise (SNR) ratios: (a) SNR = ∞, (b) SNR = 26 dB, (c) SNR = 6 dB, and (d) SNR = -6 dB.

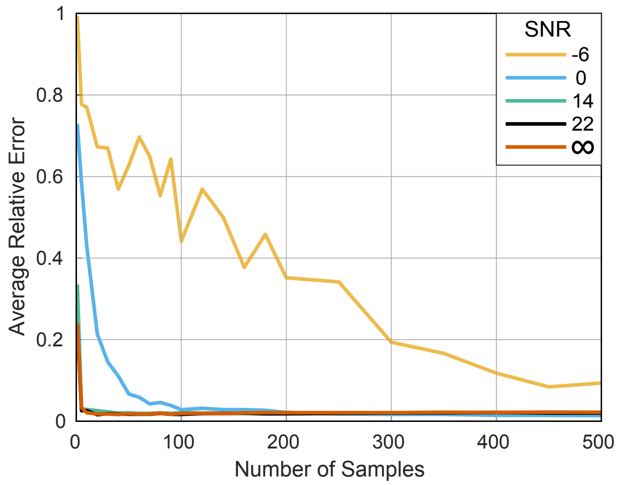

Figure S2: Relative error of estimation of the second modal frequency versus the number of trips used with simulated data. Each color represents a different SNR. In most cases, the estimation error decreases rapidly and then stabilizes at a level that represents an accuracy limit. For very high noise (SNR = -6 dB), the error decreases more slowly, requiring a larger number of bridge trips to achieve sufficient accuracy.

a random white spatio-temporal loading is applied on the bridge while the sensing vehicles are in motion [19]. Sensing vehicles are modeled as two degrees of freedom quarter-cars with linear mechanical properties, each randomly sampled from log-normal distributions with means set to the properties of a commercial vehicle. In addition, in accordance to the real sensing scenarios, vehicles adopt different constant speeds while sensing.

The resulting MPMFs are comparable to the results displayed in Figures 3 and 4 in the main text. Figure S1 shows how these estimates are effected by random measurement noise for four different SNR cases and Figure S2 shows how the error in estimating the second modal frequency decreases as the the number of bridge trips, i.e., samples increases.

Supplementary Note 4: Vehicle-Bridge-Road Interaction

The dynamics of a vehicle crossing a bridge is characterized by the interaction of three systems: the vehicle suspension system, the bridge system, and the road surface. The influence of the vehicle-bridge-road interaction (VBRI) on measurements from mobile sensor networks has been studied widely [19–30]. These studies have established the governing differential equations, constructed useful simplified models, provided informative simulations, and conducted real-world experiments, which have led to important discoveries. For example, the "vehicle driving frequency", which arises in certain vehicle scanning scenarios [21,22]; and how low vehicle speeds, stiff vehicle suspensions, and smooth road surfaces can greatly simplify VBRI models [20,23].

Most commercially available vehicles have resonant frequencies within the range of $1-3$ Hz [31,32], which may be close to modal frequencies of some short-span and medium-span bridges [21–27]. In such cases, it may be desirable to remove these unwanted vehicle vibrations as they may interfere with the identification of bridge modal properties. For instance, if only one vehicle is used for vehicle scanning data, and one of its resonance frequencies coincides with a bridge modal frequency, it is more challenging to confirm bridge modal properties - it may be necessary to incorporate data collected by another vehicle. In general, source-separation methods, e.g., blind-source [33,34], deconvolution [35–37], etc., have demonstrated an ability to remove the contributions of the vehicle suspension system from the total vehicle response, such that the remaining signal corresponds to the bridge system. The case of crowdsourcing data introduces unique stochastic elements to VBRI through the variety of vehicles and smartphones utilized. For example, when considering random vibrations caused by regular traffic on medium-span and long-span bridges, the effects of the dynamic forces induced on the bridge by the vehicle carrying the sensor are negligible [38]. In short, entropy is a desirable feature of crowdsourced vehicle-trip data which will help reduce bias in estimates of bridge properties.

The studies in this paper did not explicitly consider VBRI, yet were still successful in identifying bridge modal frequencies. The main finding here is the persistent signal strength of bridge modal frequencies among the collected data sets. In the primary application on the Golden Gate Bridge, the frequencies of interest were known and below 0.5 Hz. Therefore, downsampling and low-pass filtering eliminated vehicle dynamic effects. Free vibration tests conducted on the cars used in the controlled experiment confirmed fundamental vehicle frequencies around 1.7 Hz. The same applied to the uncontrolled ridesourcing data. The application to the short-span highway bridge (partially-controlled data) presented a situation where the bridge modal frequencies overlapped with typical vehicle modal frequencies; in which case, it may have been desirable to account for unwanted VBRI effects. However, vehicle information was not available for this data set and there was insufficient "off-bridge" driving data to confirm individual vehicle modal frequencies. Nonetheless, the fundamental modal frequency of the bridge was accurately estimated with an error that decreased as the number of datasets increased.

Future work may leverage smartphone metadata on uncontrolled data factors such as smartphone mount, or vehicle model, to help accurately characterize VBRI models. For instance, with sufficient data, acceleration measurements collected while a vehicle is *not* on the bridge, can be helpful in identifying the vehicle dynamical system, e.g., transfer functions, and quantifying sensor measurement noise. There is also a need to study the synchronization problem that arises in the case of multiple moving sensors with independent sampling properties [39–41]. Lastly, as vehicular networks emerge for urban sensing, e.g., vehicle-vehicle systems, the design of intercommunication systems should consider computation and energy costs related to data processing and transmission [42–45].

Supplementary Note 5: Impact on Bridge Service Life

How will this new wealth of information impact the longevity of existing bridges? Crowd-sourced mobile sensors will provide a nearly continuous stream of information on structural modal properties, which is vital to condition assessments and damage identification frameworks for bridges [25,46–51]. When the resulting information is incorporated into a bridge management plan, the benefits accumulate over the service life of a bridge. This section investigates the potential of crowd-sourced monitoring approaches to increase bridge service life through a structural reliability analysis [52].

A bridge owner's task is to manage degradation and allocated expenditures to ensure the bridge reaches its designed service life. Here the bridge's condition (health) is measured by the reliability index β, which is constructed by integrating the states of individual structural components. By definition, the reliability index is inversely proportional to the failure probability of the bridge. Preventive maintenance activities increase β with a factor that is proportional to the extent of the maintenance event. In a numerical setting, for a given bridge, β (i.e., β_0) are initiated in accordance with its age and current condition (based on the most recent bridge monitoring results). Then a β profile is generated over a period of time, depending on maintenance events and an inherent degradation rate. The result is called a *reliability profile*.

In a generic model of an ageing bridge, the reliability index decays over time with a rate that depends on its construction quality, its environment, its exposure to the hazards, among other factors [53]. The primary role of an SHM system is to measure samples of the reliability profile and update the status of the bridge based on actual behavior. The ultimate goal of bridge maintenance is to maximize the point in time when the reliability index crosses threshold through applying preventive maintenance actions that result in a sharp increase in the reliability profile. Simultaneously, each maintenance action has an associated cost; therefore, from an asset management perspective it is important to balance these action costs with the bridge value.

The ideal bridge management system has complete information of structural conditions at any given moment in time for the entire service life of the bridge, i.e., a continuous or finely sampled reliability profile. In practice, it is prohibitively costly to achieve this volume and rate of information using modern SHM techniques. Crowdsensed data offer an opportunity to sample structural conditions at unprecedented rates and volumes, e.g., weekly, daily, hourly, etc., which can produce a highly discretized reliability profile. To quantify the value of this discretization, the reliability profiles of distinct classes of bridges are simulated. In reliability assessments, structural failure probabilities are typically derived with respect to a computational model that is updated based on actual SHM and environmental data [54,55]. More specifically, it is assumed that the crowdsourced data enables up-to-date knowledge on structural behavior using models that accurately reflect real-world observations. Note this assumption implies access to structural dynamics information beyond modal frequencies, which would result from long-term monitoring data, advanced SHM methods, AI, computational learning, etc., as mentioned in the *Discussion* of the main text.

Reliability profiles were generated for two bridge archetypes: (a) a typical bridge in the U.S., which is 43 years old (initial reliability index, β_0, randomly sampled from $U[5, 6.4]$) and (b) a newly constructed bridge (initial β_0 randomly sampled from $U[8, 9]$). For each bridge archetype, three management policies are considered: (i) no regular inspection or preventive intervention (PI), (ii) traditional approach in which PIs occur regularly in average every 15 year as planned and to a predefined extent (e.g., bearing replacements, pavement repairs, etc.), and (iii) crowd-sourcing approach in which a one-time PI is planned based on continuous SHM information of the bridge status and applied when the status reaches the service limit (a bridge is out of service when β falls below 4.6).

For simplicity, the installation and maintenance costs of the sensor networks in policy (iii) are not considered. Note, the cost of monitoring in the crowd-sourcing framework (policy ii) is very low comparatively because the devices have already been purchased and the necessary mobility patterns are already in place. For an equal comparison, the maintenance parameters are adjusted such that the present value of the maintenance cost is identical between policies (ii) and (iii). For each bridge archetype, the reliability modeling parameters such as initial reliability index β_0, degrading rate, and maintenance-caused improvement are randomly selected from distinct distributions as recommended in [56] and $10,000$ reliability profiles are generated.

In Figure S3, the average reliability profiles under different policies are plotted plus or minus one standard deviation (shaded areas). The averaged profiles (solid lines) show the expected values for the reliability index. The intersection of the solid line and the service limit indicates the expected service life of the bridge. These values are highlighted in bold on each part of Figure S3. The results differ significantly between aged and newly constructed bridges regardless of the management plan. In both bridge archetypes, continuous monitoring enabled timely maintenance that extended the service life of a bridge compared to current methods without additional costs. On average, crowd-sourced data added 2.6 years of service to a typical bridge (a 15% increase). The most substantial benefit was observed for a newly constructed bridge, whose service was extended by 14.7 years (a 30% increase).

These analyses are the first to quantify the cumulative benefits provided by frequent, large-scale bridge health monitoring. The continuous SHM information utilized to assess a one-time PI, would be based on long-term bridge health data including modal frequencies and other structural damage metrics. Modal frequencies are a gateway to determining additional modal properties (mode shapes, damping ratios, and higher modes) and structural features which have explicit relationships with certain types of bridge damage and deterioration, e.g., mode shape curvature, local stiffness, etc. [28,46–48,57,58]. Such "damage-sensitive features" are important in the context of long-term structural behavior because large fluctuations, e.g., $5-10\%$, of a bridge's modal frequency can be normal in some climates however do not

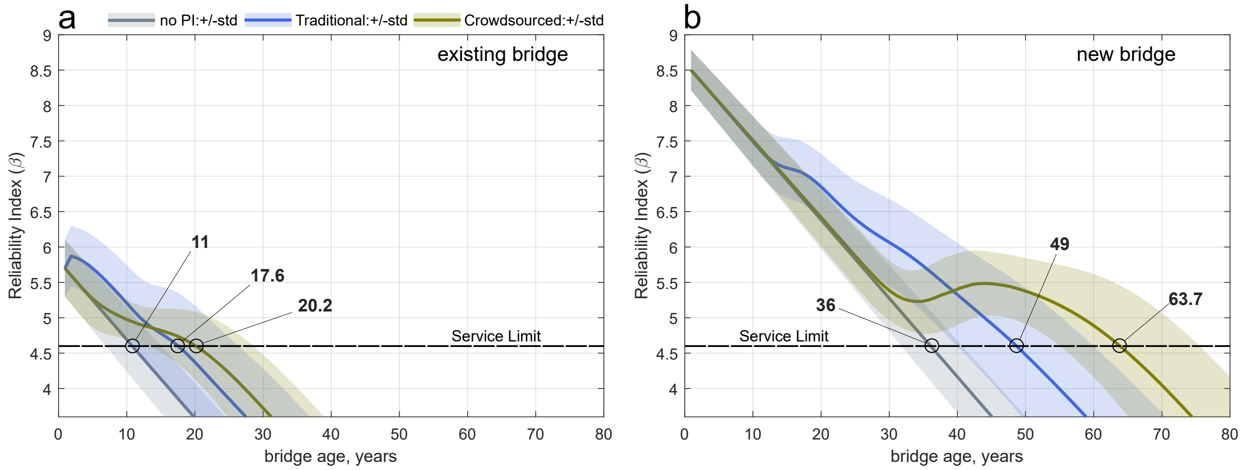

Figure S3: Reliability profiles of bridges based on Monte Carlo analyses. a) existing bridge at a typical age (43 years old); b) newly constructed bridge. Three policies are considered for each bridge archetype which have different effects on the bridge service life: (i) no preventive intervention (PI), (ii) traditional approach in which PI occurs in 15-year intervals, and (iii) crowd-sourcing approach in which a one-time PI is planned based on crowd-sourced information of the bridge status. In each case, the line represents the mean value and the shaded area indicates confidence intervals of plus or minus one standard deviation. The bridge service life ends when the reliability index falls below the service limit ($\beta = 4.6$). The expected value for service life is highlighted by a circle marker with its corresponding value in bold. The crowdsourcing policy extends the service life of both bridge archetypes (regardless of the initial state). Note the life extension is significantly higher when the policy is adopted at the beginning of a bridge's operation: 14.5 year lifespan increase (30%) for new bridges vs. 2.5 year lifespan increase (15%) for typical bridges.

necessarily represent a change in structural condition. Recent studies have successfully leveraged the environmental co-dependencies of modal frequencies to predict expected trends and detect unexpected ones based on as little as eight months of data [59]. For instance the changes in modal frequencies not explained by environmental data have been linked to bridge damage in real-world scenarios [48,59–65].

Supplementary References

[1] Anil K Chopra. *Dynamics of structures: theory and applications to earthquake engineering.* Prentice-Hall, 2001.

[2] Ahmed M Abdel-Ghaffar. Dynamic analyses of suspension bridge structures. Technical report, Earthquake Engineering Research Laboratory, California Institute of Technology, 1976.

[3] Ahmed M Abdel-Ghaffar and George W Housner. Ambient vibration tests of suspension bridge. *Journal of the Engineering Mechanics Division*, 104(5):983–999, 1978.

[4] Ahmed M Abdel-Ghaffar and Robert H Scanlan. Ambient vibration studies of golden gate bridge: I. suspended structure. *Journal of Engineering Mechanics*, 111(4): 463–482, 1985.

[5] Julius S Bendat and Allan G Piersol. Engineering applications of correlation and spectral analysis. *New York*, 1980.

[6] Julius S Bendat and Allan G Piersol. *Random data: analysis and measurement procedures*, volume 729. John Wiley & Sons, 2011.

[7] George H James III, Thomas G Carne, and James P Lauffer. The natural excitation technique (next) for modal parameter extraction from operating wind turbines. *NASA STI/Recon Technical Report N*, 93:28603, 1993.

[8] Rune Brincker, Lingmi Zhang, and Palle Andersen. Modal identification of output-only systems using frequency domain decomposition. *Smart materials and structures*, 10(3):441, 2001.

[9] Olivier Rioul and Martin Vetterli. Wavelets and signal processing. *IEEE signal processing magazine*, 8 (ARTICLE):14–38, 1991.

[10] Norden E Huang, Zheng Shen, Steven R Long, Manli C Wu, Hsing H Shih, Quanan Zheng, Nai-Chyuan Yen, Chi Chao Tung, and Henry H Liu. The empirical mode decomposition and the hilbert spectrum for nonlinear and non-stationary time series analysis. *Proceedings of the Royal Society of London. Series A: Mathematical, Physical and Engineering Sciences*, 454(1971):903–995, 1998.

[11] Patrick Flandrin, Gabriel Rilling, and Paulo Goncalves. Empirical mode decomposition as a filter bank. *IEEE signal processing letters*, 11(2):112–114, 2004.

[12] TACM Claasen and WFG Mecklenbrauker. The wigner distribution—a tool for time-frequency signal analysis. *Philips J. Res*, 35(3):217–250, 1980.

[13] Ingrid Daubechies. A nonlinear squeezing of the continuous wavelet transform based on auditory nerve models. *Wavelets in medicine and biology*, pages 527–546, 1996.

[14] Ingrid Daubechies, Jianfeng Lu, and Hau-Tieng Wu. Synchrosqueezed wavelet transforms: An empirical mode decomposition-like tool. *Applied and computational harmonic analysis*, 30(2):243–261, 2011.

[15] Gaurav Thakur, Eugene Brevdo, Neven S Fučkar, and Hau-Tieng Wu. The synchrosqueezing algorithm for time-varying spectral analysis: Robustness properties and new paleoclimate applications. *Signal Processing*, 93(5):1079–1094, 2013.

[16] Qingtang Jiang and Bruce W Suter. Instantaneous frequency estimation based on synchrosqueezing wavelet transform. *Signal Processing*, 138:167–181, 2017.

[17] Pierre Goupillaud, Alex Grossmann, and Jean Morlet. Cycle-octave and related transforms in seismic signal analysis. *Geoexploration*, 23(1):85–102, 1984.

[18] Shamim N Pakzad, Gregory L Fenves, Sukun Kim, and David E Culler. Design and implementation of scalable wireless sensor network for structural monitoring. *Journal of infrastructure systems*, 14(1):89–101, 2008.

[19] Soheil Sadeghi Eshkevari, Thomas J Matarazzo, and Shamim N Pakzad. Simplified vehicle–bridge interaction for medium to long-span bridges subject to random traffic load. *Journal of Civil Structural Health Monitoring*, pages 1–15, 2020.

[20] Reto Cantieni. Dynamic behavior of highway bridges under the passage of heavy vehicles. 1992.

[21] Yeong-Bin Yang and Bing-Houng Lin. Vehicle-bridge interaction analysis by dynamic condensation method. *Journal of Structural Engineering*, 121(11):1636–1643, 1995.

[22] Yeong-Bin Yang and Jong-Dar Yau. Vehicle-bridge interaction element for dynamic analysis. *Journal of Structural Engineering*, 123(11):1512–1518, 1997.

[23] Sara Barella and Reto Cantieni. Vehicle/bridge interaction for medium span bridges-research element 6 of the oecd ir6 divine project. In *Proc., 4th Int. Symp. Heavy Veh. Weights Dimensions Road Transp. Technol.*, pages 355–364, 1995.

[24] Jann N Yang, Yu Lei, S Lin, and N Huang. Hilbert-huang based approach for structural damage detection. *Journal of engineering mechanics*, 130(1):85–95, 2004.

[25] Y-B Yang, CW Lin, and JD Yau. Extracting bridge frequencies from the dynamic response of a passing vehicle. *Journal of Sound and Vibration*, 272(3-5):471–493, 2004.

[26] CW Lin and YB Yang. Use of a passing vehicle to scan the fundamental bridge frequencies: An experimental verification. *Engineering Structures*, 27(13):1865–1878, 2005.

[27] YB Yang and KC Chang. Extracting the bridge frequencies indirectly from a passing vehicle: Parametric study. *Engineering Structures*, 31(10):2448–2459, 2009.

[28] Arturo González, Eugene J OBrien, and PJ McGetrick. Identification of damping in a bridge using a moving instrumented vehicle. *Journal of Sound and Vibration*, 331(18):4115–4131, 2012.

[29] Dionysius M Siringoringo and Yozo Fujino. Estimating bridge fundamental frequency from vibration response of instrumented passing vehicle: analytical and experimental study. *Advances in Structural Engineering*, 15 (3):417–433, 2012.

[30] YB Yang, MC Cheng, and KC Chang. Frequency variation in vehicle–bridge interaction systems. *International Journal of Structural Stability and Dynamics*, 13(02): 1350019, 2013.

[31] Maurice Olley. Independent wheel suspension—its whys and wherefores. *SAE Transactions*, pages 73–81, 1934.

[32] DA Crolla and RP King. Olley's "flat ride" revisited. *Vehicle System Dynamics*, 33(sup1):762–774, 1999.

[33] J-F Cardoso. Blind signal separation: statistical principles. *Proceedings of the IEEE*, 86(10):2009–2025, 1998.

[34] Fabien Poncelet, Gaëtan Kerschen, J-C Golinval, and Damien Verhelst. Output-only modal analysis using blind source separation techniques. *Mechanical systems and signal processing*, 21(6):2335–2358, 2007.

[35] William F Milliken, Douglas L Milliken, and Maurice Olley. *Chassis design: principles and analysis*, volume 400. Society of Automotive Engineers Warrendale, 2002.

[36] Patrick J McGetrick, Chul-Woo Kim, Arturo González, and Eugene JO Brien. Experimental validation of a drive-by stiffness identification method for bridge monitoring. *Structural Health Monitoring*, 14(4):317–331, 2015.

[37] Soheil Sadeghi Eshkevari, Thomas J. Matarazzo, and Shamim N. Pakzad. Bridge modal identification using acceleration measurements within moving vehicles. *Mechanical Systems and Signal Processing*, 141:106733, 2020. ISSN 0888-3270. doi: https://doi.org/10.1016/j.ymssp.2020.106733.

[38] Soheil Sadeghi Eshkevari, Liam Cronin, Shamim N Pakzad, and Thomas J Matarazzo. Bridge structural health monitoring using asynchronous mobile sensing data. *arXiv preprint arXiv:2007.09249*, 2020.

[39] Farokh Marvasti. *Nonuniform sampling: theory and practice*. Springer Science & Business Media, 2012.

[40] Flavio Zabini, Alex Calisti, Davide Dardari, and Andrea Conti. Random sampling via sensor networks: Estimation accuracy vs. energy consumption. In *2016 24th European Signal Processing Conference (EUSIPCO)*, pages 130–134. IEEE, 2016.

[41] Flavio Zabini and Andrea Conti. Inhomogeneous poisson sampling of finite-energy signals with uncertainties in r^d. *IEEE Transactions on Signal Processing*, 64(18): 4679–4694, 2016.

[42] Z Cihan Taysi and A Gokhan Yavuz. Routing protocols for geonet: A survey. *IEEE Transactions on Intelligent Transportation Systems*, 13(2):939–954, 2012.

[43] Ehsan Rabiei, Uwe Haberlandt, Monika Sester, and Daniel Fitzner. Rainfall estimation using moving cars as rain gauges–laboratory experiments. *Hydrology and Earth System Sciences*, 17(11):4701–4712, 2013.

[44] Alessandro Bazzi, Barbara M Masini, Alberto Zanella, and Gianni Pasolini. Ieee 802.11 p for cellular offloading in vehicular sensor networks. *Computer Communications*, 60:97–108, 2015.

[45] Emanuele Massaro, Chaewon Ahn, Carlo Ratti, Paolo Santi, Rainer Stahlmann, Andreas Lamprecht, Martin Roehder, and Markus Huber. The car as an ambient sensing platform [point of view]. *Proceedings of the IEEE*, 105(1):3–7, 2016.

[46] Charles R Farrar and David Jauregui. *Damage detection algorithms applied to experimental and numerical modal data from the I-40 bridge.* Los Alamos National Laboratory, 1996.

[47] MM Abdel Wahab and Guido De Roeck. Damage detection in bridges using modal curvatures: application to a real damage scenario. *Journal of Sound and vibration*, 226(2):217–235, 1999.

[48] Bart Peeters and Guido De Roeck. One-year monitoring of the z24-bridge: environmental effects versus damage events. *Earthquake engineering & structural dynamics*, 30(2):149–171, 2001.

[49] K Krishnan Nair, Anne S Kiremidjian, and Kincho H Law. Time series-based damage detection and localization algorithm with application to the asce benchmark structure. *Journal of Sound and Vibration*, 291(1-2): 349–368, 2006.

[50] Ruigen Yao and Shamim N Pakzad. Autoregressive statistical pattern recognition algorithms for damage detection in civil structures. *Mechanical Systems and Signal Processing*, 31:355–368, 2012.

[51] EJ Cross, KY Koo, JMW Brownjohn, and K Worden. Long-term monitoring and data analysis of the tamar bridge. *Mechanical Systems and Signal Processing*, 35 (1-2):16–34, 2013.

[52] Dan M Frangopol, Kai-Yung Lin, and Allen C Estes. Life-cycle cost design of deteriorating structures. *Journal of structural engineering*, 123(10):1390–1401, 1997.

[53] PBR Dissanayake and PAK Karunananda. Reliability index for structural health monitoring of aging bridges. *Structural Health Monitoring*, 7(2):175–183, 2008.

[54] Hui Li, Shunlong Li, Jinping Ou, and Hongwei Li. Reliability assessment of cable-stayed bridges based on structural health monitoring techniques. *Structure and Infrastructure Engineering*, 8(9):829–845, 2012.

[55] A Baldomir, I Kusano, S Hernandez, and JA Jurado. A reliability study for the messina bridge with respect to flutter phenomena considering uncertainties in experimental and numerical data. *Computers & Structures*, 128:91–100, 2013.

[56] Dan M Frangopol, Jung S Kong, and Emhaidy S Gharaibeh. Reliability-based life-cycle management of highway bridges. *Journal of computing in civil engineering*, 15(1):27–34, 2001.

[57] AK Pandey and M Biswas. Damage detection in structures using changes in flexibility. *Journal of sound and vibration*, 169(1):3–17, 1994.

[58] M Ruzzene, A Fasana, L Garibaldi, and B Piombo. Natural frequencies and dampings identification using wavelet transform: application to real data. *Mechanical systems and signal processing*, 11(2):207–218, 1997.

[59] Yabin Liang, Dongsheng Li, Gangbing Song, and Qian Feng. Frequency co-integration-based damage detection for bridges under the influence of environmental temperature variation. *Measurement*, 125:163–175, 2018.

[60] Jong Jae Lee, Jong Won Lee, Jin Hak Yi, Chung Bang Yun, and Hie Young Jung. Neural networks-based damage detection for bridges considering errors in baseline finite element models. *Journal of Sound and Vibration*, 280(3-5):555–578, 2005.

[61] MELİN Sahin and RA Shenoi. Quantification and localisation of damage in beam-like structures by using artificial neural networks with experimental validation. *Engineering structures*, 25(14):1785–1802, 2003.

[62] Marian Ralbovsky, Stefan Deix, and Rainer Flesch. Frequency changes in frequency-based damage identification. *Structure and Infrastructure Engineering*, 6(5): 611–619, 2010.

[63] Jeong-Tae Kim, Jae-Hyung Park, and Byung-Jun Lee. Vibration-based damage monitoring in model plate-girder bridges under uncertain temperature conditions. *Engineering Structures*, 29(7):1354–1365, 2007.

[64] Wei Fan and Pizhong Qiao. Vibration-based damage identification methods: a review and comparative study. *Structural health monitoring*, 10(1):83–111, 2011.

[65] Chenhao Jin, Shinae Jang, Xiaorong Sun, Jingcheng Li, and Richard Christenson. Damage detection of a highway bridge under severe temperature changes using extended kalman filter trained neural network. *Journal of Civil Structural Health Monitoring*, 6(3):545–560, 2016.
